# Interacting plexcitons for designed ultrafast optical nonlinearity in a monolayer semiconductor

**DOI:** 10.1038/s41377-022-00754-3

**Published:** 2022-04-14

**Authors:** Yuxiang Tang, Yanbin Zhang, Qirui Liu, Ke Wei, Xiang’ai Cheng, Lei Shi, Tian Jiang

**Affiliations:** 1 College of Advanced Interdisciplinary Studies, National University of Defense Technology, 410073 Changsha, China; 2 Key Laboratory of Micro- and Nano-Photonic Structures (Ministry of Education), and State Key Laboratory of Surface Physics, Department of Physics, Fudan University, 200433 Shanghai, China; 3 State Key Laboratory of High Performance Computing, College of Computer, National University of Defense Technology, 410073 Changsha, China; 4 Beijing Institute for Advanced Study, National University of Defense Technology, 100000 Beijing, China

**Keywords:** Polaritons, Nonlinear optics, Ultrafast photonics

## Abstract

Searching for ideal materials with strong effective optical nonlinear responses is a long-term task enabling remarkable breakthroughs in contemporary quantum and nonlinear optics. Polaritons, hybridized light-matter quasiparticles, are an appealing candidate to realize such nonlinearities. Here, we explore a class of peculiar polaritons, named plasmon–exciton polaritons (plexcitons), in a hybrid system composed of silver nanodisk arrays and monolayer tungsten-disulfide (WS_2_), which shows giant room-temperature nonlinearity due to their deep-subwavelength localized nature. Specifically, comprehensive ultrafast pump–probe measurements reveal that plexciton nonlinearity is dominated by the saturation and higher-order excitation-induced dephasing interactions, rather than the well-known exchange interaction in traditional microcavity polaritons. Furthermore, we demonstrate this giant nonlinearity can be exploited to manipulate the ultrafast nonlinear absorption properties of the solid-state system. Our findings suggest that plexcitons are intrinsically strongly interacting, thereby pioneering new horizons for practical implementations such as energy-efficient ultrafast all-optical switching and information processing.

## Abstract (web summary)

We demonstrate that plexcitons in monolayer semiconductors sustain giant nonlinearity at room temperature and thus very hopeful to realize the practical implementation of polaritonic devices.

## Graphical abstract

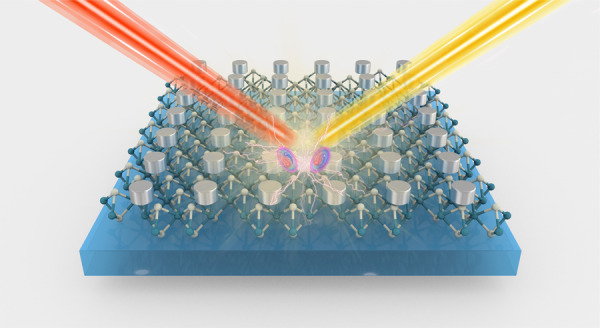

## Introduction

Polaritons are half-light half-matter quasiparticles resulting from the resonance coupling between electromagnetic waves and elementary excitations^1^. As a typical representative of these coherent superposition states, microcavity exciton polaritons (also referred to as microcavity polaritons here) are formed by quantum hybridization of semiconductor excitons and microcavity photons^2^. Benefitting from their mixed light and matter nature, microcavity polaritons have yielded many observations on collective quantum effects, such as Bose–Einstein condensation^3^, superfluidity^4^, and nonclassical light^5,6^, and can be engineered for a wealth of technological applications, including low-threshold polariton lasers^7^, all-optical logic circuits^8^, and even ultrafast optical switches at the single-photon limit^9^. Of these fascinating phenomena, the intrinsic strong nonlinearity inherited from the exciton component is the key to most of the distinctive physical features of polaritons^10,11^. However, in traditional Wannier-Mott and Frenkel semiconductor materials with hydrogenic-type excitonic states, the polariton nonlinearity and exciton-photon coupling strength are two quantities with an evitable trade-off, as the polariton nonlinearity scales with the exciton Bohr radius, while the coupling strength is inversely proportional to the exciton Bohr radius. Currently, the highest polariton nonlinearity is usually achieved in GaAs^12,13^ and CdTe^14^ microcavities with large Bohr radii, but operation at cryogenic temperatures is required due to their small exciton binding energy. Instead, robust room-temperature exciton-photon coupling strength can be realized in ZnO^15^ and organic systems^16^ with large exciton binding energy (small Bohr radii), but often at the expense of weak polariton nonlinearity. This contradiction severely limits the development of room-temperature polaritonic devices, in which sufficiently strong nonlinearity and coupling strength are simultaneously required.

Recently emerged monolayer (ML) transition metal dichalcogenides (TMDs) offer a unique solution to this trade-off due to their modified Coulomb potential beyond the hydrogenic picture^17^, which essentially improves the exciton nonlinearity despite the ~1 nm exciton Bohr radius^18–21^. Meanwhile, successful demonstrations of polariton nonlinearity enhancement by utilizing trions^22,23^, polarons^24^, Rydberg excitons^25^, and moiré excitons^26^ in TMDs further demonstrate their capacity for room-temperature polaritonic applications. Nonetheless, such an increase in nonlinearity still involves the risk of instability and sophisticated fabrication of samples.

One strategy for higher room-temperature nonlinearity in TMD-based polaritons without compromising the stability is to squeeze them into very tiny, deep-subwavelength areas to achieve adequately strong interactions. Plasmon–exciton polaritons (i.e., plexcitons)^27–30^, as a special example of hybrid polaritons constructed by plasmon–exciton coupled modes^31^, are naturally localized in their spatial distribution as they adopt highly plasmon-like charge and field characteristics^32^, and thus very hopeful to reach this prospect. Although robust coupling strengths in TMD-based plexcitons have been intensively reported^33–38^, research on their nonlinearity remains scarce^39^, and the fundamental issue of whether the nanoscale confinement nature will impart plexcitons with superior nonlinearity remains ambiguous.

Here, we systematically study the plexciton nonlinearity in a Ag nanodisk (ND)-ML WS_2_ hybrid sample. By applying femtosecond pump–probe measurements, we first reveal the subtle photophysical dynamics of the hybrid sample, including coherent plexcitons, incoherent plasmon/exciton populations, and heat effect processes. Then, we provide experimental evidence of nontrivial higher-order nonlinearity in plexcitons arising from the excitation-induced dephasing effect of their exciton component. Combining this giant nonlinear interaction with the great tunability of plexcitons, we finally implement active design of ultrafast nonlinear absorption responses in plexcitons at comparatively low excitation energy, which highlights the great potential of plexcitons in nonlinear optical applications.

## Results

### Emergence of plexcitons in Ag ND–WS_2_ hybrid systems

**Fig. 1:**
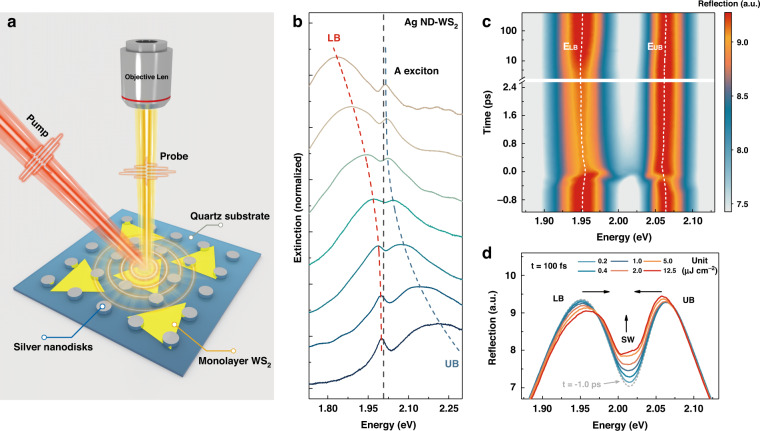
Ag ND–WS_2_ plexcitonic system and its steady-state/ultrafast optical properties. **a** Schematic illustration of the Ag ND–WS_2_ hybrid system, in which time-resolved pump–probe microscopy was adopted to study its ultrafast optical responses. **b** Normalized extinction spectra of the Ag ND–WS_2_ hybrid system with increasing disk diameter (from bottom to top) in the range of 80–140 nm, with an increment of ~10 nm. The red and blue dashed lines represent the UB and LB of plexcitons, respectively, and the black dashed line indicates the exciton resonance location. **c** Time-resolved reflection spectra scan of the zero-detuning (disk diameter of 110 nm) Ag ND–WS_2_ plexcitonic system under 2.0 eV pump excitation conditions at an incident fluence of 5 µJ cm^−2^. The inserted white dashed line indicates the peak energy evolution of UB and LB plexcitons as a function of time. **d** Reflection spectra of the Ag ND–WS_2_ plexcitonic system projected from the cross-section of (**c**) at *t* = 100 fs. From bottom to top, the incident fluence increases from 0.2 µJ cm^−2^ to 12.5 µJ cm^−2^. The grey dashed line is the probe reflection spectrum at −1 ps, which is unaffected by the pump pulse

A schematic diagram of the sample configuration in this work is shown in Fig. 1a, where plasmonic arrays composed of disk-shaped silver nanoparticles with a height of 30 nm and a lattice period of 300 nm were directly patterned onto ML WS_2_ flakes supported by a quartz substrate. A thin film (200 nm) of polymethyl methacrylate (PMMA) was then used to cover the surface of the structure to avoid unwanted degradation (see Supplementary Note 1 for more details regarding sample fabrication and characterization). An atomically thin TMD semiconductor, ML WS_2_, was chosen as the resonant absorbing medium because it possesses pronounced excitonic oscillator strength and a narrow absorption band at room temperature (Fig. S1c), facilitating the investigation into coherent plexcitonic interactions. For the periodically arranged Ag NDs applied here, two different kinds of modes are excited upon illumination^40^. One is the localized surface plasmon resonance (LSPR) induced by strong collective surface-bound oscillations of mobile charges in individual metallic ND, and the other is the delocalized diffractive state that arises from periodic lattices satisfying the Bragg scattering condition, also known as Rayleigh anomalies (RAs). These two modes can effectively interact with each other under certain conditions, giving rise to a new hybrid plasmonic/photonic state, i.e., plasmonic surface lattice resonance (SLR). However, the influence of the diffractive state is undesired in our study, as it might change the localized nature (subwavelength mode confinement) of the LSPR to the delocalized nature (micrometre-scale delocalization) of the SLR. Therefore, RAs were designed to depart from the flat band of the LSPR in the frequency domain (by adjusting the lattice period; see Fig. S2 for dispersions). This means that the Ag ND plasmonic arrays herein behave similar to a single independent nanostructure, in which the electromagnetic coupling effect (both near-field and far-field) between individual NDs can be nearly neglected.

The emergence of plexcitons in the Ag ND–WS_2_ hybrid system was first verified, as shown in Fig. 1b, via extinction measurements (see “Methods”), in which the spectral location of the LSPR (${{{{E}}}}_{{{{\mathrm {p}}}}}$) was gradually tuned through the exciton resonance (${{{{E}}}}_{{{\mathrm{x}}}}$) of WS_2_ by adjusting the diameters of the NDs from 80 nm to 140 nm in an increment of ~10 nm. Two resultant energy branches, namely, the upper branch (UB) and lower branch (LB) of plexcitons, demonstrate a clear anticrossing behaviour near the position of the WS_2_ exciton state, indicating valid formation of plasmon–exciton hybridization. To further ascertain the origins of hybridization (Fano interference or mode splitting) and clarify the coupling strength of the plasmon–exciton interaction, coupled oscillator model^41^ (COM, see Eq. (1) in “Methods”) analysis was used to fit the experimental spectrum under the zero detuning condition (*E*_p_ = *E*_x_). The corresponding fitting results and parameters are presented in Fig. S3 and Table S1, which yield a considerable calculated coupling strength (*g*) between the plasmon and exciton of 92.6 meV. Note that the curve used for COM analysis is the reflection spectrum acquired by time-resolved measurements rather than the extinction spectrum from steady-state measurements (the reasons for the spectral differences between reflection and extinction measurements are thoroughly discussed in Supplementary Note 2.2). By comparing the extracted g with the linewidth (i.e., dissipation rate) of the plasmon (${\upgamma}_{{{\mathrm{p}}}} = 270\;{{{\mathrm{meV}}}}$) and exciton (${\upgamma}_{{{\mathrm{x}}}} = 60\;{{{\mathrm{meV}}}}$), we found that *g* is almost equal to $({\upgamma}_{{{\mathrm{p}}}} - {\upgamma}_{{{\mathrm{x}}}})/2$ and far beyond ${\upgamma}_{{{\mathrm{x}}}}$, which suggests that both Fano interference (${{{{g}}}} \,<\, ({\upgamma}_{{{\mathrm{p}}}} - {\upgamma}_{{{\mathrm{x}}}})/2$) and mode splitting (${{{{g}}}} \,>\, ({\upgamma}_{{{\mathrm{p}}}} - {\upgamma}_{{{\mathrm{x}}}})/2$) should contribute to the hybridization between the plasmon and exciton here. This numerical result, together with the experimental appearance of anticrossing, indicates that plexcitons formed in our system are in the intermediate coupling regime (i.e., on the border of Fano interference and mode splitting). At this moment, spectral splitting is already apparent in the frequency domain, but the Rabi oscillations in the time domain are still obscured by the large damping of the plasmonic mode. This very interesting moderate regime has been extensively reported in recent years, especially for plasmon/exciton hybrid systems^36,38,42–44^, and bridges the widely unexplored gap between weak and strong coupling regimes, making plexcitons essentially different from traditional microcavity polaritons.

### Ultrafast photophysical dynamics of plexcitons

Before shifting to the investigation of plexciton nonlinearity, a major task that needs to be first addressed is to figure out the complex photophysical dynamics of plexcitons, in particular the characteristic lifetimes of coherent plexcitons, the incoherent plasmon/exciton populations, and the heat effect, which ultimately determine the exact time at which nonlinearity can be probed. Time-resolved pump–probe measurements (see the “Methods” section for additional details) were thus performed under resonant excitation conditions (pump energy of 2.0 eV) to detect the ultrafast responses of Ag ND–WS_2_ plexcitonic sample at zero detuning and a further comparison of the dynamics of Ag ND–WS_2_ hybrid system with its isolated WS_2_ and Ag ND constituent is also illustrated in Supplementary Note 3. Figure 1c shows a typical recording result of probe reflection as a function of time delay at an incident fluence of 5.0 µJ cm^−2^, and the two white dashed lines (marked by *E*_UB_ and *E*_LB_) represent the temporal evolution of the peak energy for UB and LB plexciton resonance, respectively (more results at various fluences are available in Fig. S5). Notably, to accurately extract the peak energy of UB and LB plexcitons, the influence of spectral oscillations generated by the transient grating effect (throughout the pump–probe pulse overlapping duration) should be carefully considered and excluded (see Supplementary Note 4.2 for details). The temporal evolutions of *E*_UB_ and *E*_LB_ are found to exhibit entirely different behaviours, which can be observed more clearly in Fig. S7a. *E*_UB_ initially exhibits a significant redshift right after pump excitation and then slowly return to equilibrium state in dozens of ps. In contrast, *E*_LB_ starts with a sharp blueshift and rapidly turns into a redshift at hundreds of femtoseconds, then the redshift gradually increases to a maximum at several ps and finally returns to equilibrium state within tens of ps. These striking distinctions between the time evolutions of *E*_UB_ and *E*_LB_ strongly imply that the optical responses of plexcitons at different time scales are governed by different physical mechanisms. Specifically, the probe reflection of plexcitons is expected to show nonlinear responses due to^24,45^ (i) the presence of pump-induced coherent plexcitons, (ii) the perturbation from the pump-induced incoherent plasmon/exciton populations, and (iii) the modification from the pump-induced heat effect.

To identify the unique occurrence time scales of these three physical processes, we carry out basic processing on *E*_UB_ and *E*_LB_ by simply adding (*E*_UB_ + *E*_LB_) and subtracting (*E*_UB_ − *E*_LB_) them at every time delay, as displayed in Fig. S7b. According to Eq. (2) in the “Methods”, the value of *E*_UB_ − *E*_LB_ (defined as the Rabi splitting, ${{{\mathrm{{\Omega}}}}}$) directly monitors the coupling strength between the plasmon and exciton. Therefore, its temporal evolution (left column of Fig. S7b) can be utilized to reflect the united dynamics of coherent plexcitons and the incoherent plasmon/exciton populations since the coupling strength will be altered as long as photogenerated excitations exist^46^. The recovery time of the splitting after optical stimulus is fitted to be approximately 0.6 ps, revealing the intrinsic ultrafast coherent and incoherent decay of plexcitonic systems. In fact, coherent plexciton relaxation is supposed to be much faster (approximately at the tens of femtoseconds level) and to manifest as several strong Rabi oscillations (in the strong coupling regime)^47^ or a long-lasting coherent tail (in the intermediate coupling regime)^48^ in the time domain after excitation. Unfortunately, these fine features of the coherent process cannot be distinguished in the measurements because of the limited time resolution (~200 fs) of our pump–probe techniques. Consequently, the splitting recovery time showcased here mainly represents the lifetime of subsequent incoherent plasmon/exciton population relaxation, which is referred to as the stage where coherence is lost in plexcitons and the energy stored in incoherent excitons dissipates very quickly via incoherent energy transfer to plasmons^49,50^. In addition, we study the dynamics of the heat effect in plexcitonic system by tracing the time evolution of *E*_UB_ + *E*_LB_ (right column of Fig. S7b), as its value reflects the sum of the energy of plasmon and exciton resonances (*E*_p_ + *E*_x_) according to Eq. (3) in “Methods”. As can be observed, the dynamics of *E*_UB_ + *E*_LB_ shows a continuous redshift for the whole time delay scan and gradually reach a peak at almost 5 ps. We attribute this redshift to the band renormalization arising from the heating effect in plexcitonic systems, which can be interpreted as the pump-excited plasmons rapidly converting the absorbed photon energy into heat, resulting in remarkable local thermal accumulation in Ag NDs. Later, the heat transfers across the interface and raises the temperature of the adjacent WS_2_ lattice, leading to an obvious redshift of ${{{\mathrm{E}}}}_{{{\mathrm{x}}}}$^51^. Here, the heat-induced energy shift of *E*_P_ can be neglected since individual Ag NDs show barely any transient response under this incident fluence condition, as displayed in Fig. S9. The overall physical picture of the plexcitons after pulsed optical excitation is schematically summarized in Fig. S8, which explicitly unveils cascade-like relaxation processes of plexcitons.

In principle, the nonlinearity of plexcitons must be studied within their coherent lifetime. Therefore, only probe reflection at or slightly after the pump excitation duration can be utilized to embody the nonlinear interaction of plexcitons. In this work, the probe reflection at *t* = 100 fs is compromisingly selected (time zero is defined in the “Methods”) since at this moment, the photogenerated coherent plexcitons are still partly preserved and the effect from the transient grating imposed by the excitation pulse has completely disappeared. In addition, the roles of the incoherent plasmon/exciton population and heat effect can also be ignored at *t* = 100 fs, as they function primarily at a later time. Figure 1d presents a series of reflection spectra of coherent plexcitons at 100 fs under various pump incident fluences. Two distinctive features are observed: with increasing incident fluence density, (i) the UB and LB plexcitons symmetrically shift towards the exciton resonance, resulting in reduction of spectral splitting, and (ii) the UB and LB plexcitons exhibit considerable linewidth broadening, bringing about an apparent rise of the splitting window (SW). These observations suggest that plexcitons strongly interact with each other, and the origins of these plexciton nonlinearities will be thoroughly discussed in the next section.

### Giant nonlinear interaction of plexcitons

**Fig. 2:**
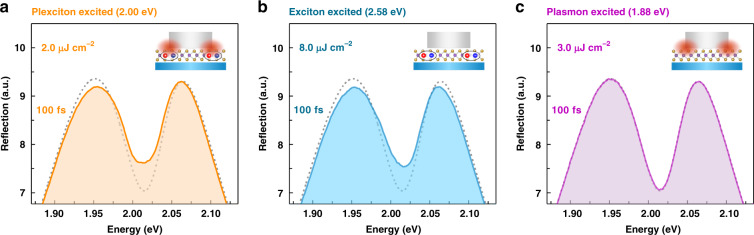
Selective excitation of plasmonic/excitonic components in plexcitons. Reflection spectra of the Ag ND–WS_2_ plexcitonic system at 100 fs time delay under (**a**) plexcitonic excitation (2.0 eV), (**b**) excitonic excitation (2.58 eV), and (**c**) plasmonic excitation (1.88 eV). To ensure the same fluences absorbed by the sample in each pump excitation situation (roughly estimated by the value of the incident fluence multiplied by the extinction coefficient), the incident fluences for pump excitation at 2.0 eV, 2.58 eV, and 1.88 eV are tailored to be 2.0 µJ cm^−2^, 8.0 µJ cm^−2^, and 3.0 µJ cm^−2^, respectively. The grey dashed lines are the plexciton reflection spectrum unaffected by the pump excitation, and the inserted schematics show the components excited in the plexcitons

Different from microcavity polaritons, whose nonlinearity exclusively comes from their excitonic component, as cavity photons do not interact with one another^52^, the nonlinearity of plexcitons might have an extra source from their plasmonic component in addition to the excitonic component. Therefore, identifying the role played by the excitonic and plasmonic components in plexciton nonlinearity is of foremost importance, which is a prerequisite to specify the type of plexcitonic nonlinear interaction. For this reason, we selectively excited the ML WS_2_ (i.e., excitons) and Ag ND (i.e., plasmons) elements in the zero-detuning Ag ND–WS_2_ sample and simultaneously monitored the ultrafast optical response of the whole hybrid system. Specifically, a pump pulse with an energy of 2.58 eV was chosen to selectively excite the excitonic component of plexcitons since it can induce interband transition in ML WS_2_ semiconductors but it is off resonant with the plasmon resonance^50^, and a pump pulse with an energy of 1.88 eV was employed to only excite the plasmonic component of plexcitons, as it cannot excite optical bandgap of the ML WS_2_ but it is within the broad linewidth of plasmon resonance. Moreover, for the case of resonant pump excitation (i.e., 2.0 eV), both the excitonic and plasmonic components of plexcitons were stimulated. Relevant experimental results of the plexciton reflection spectra at 100 fs under these excitation energies are compared in Fig. 2, where the absorbed fluences for each pump excitation were adapted to be the same to ensure nearly equal populations of photoinjected plexcitons, excitons, and plasmons. Interestingly, the measured plexciton reflection spectra under 2.0 eV (plexciton) and 2.58 eV (exciton) excitation conditions are observed to be almost identical (Fig. 2a, b) despite some minor differences, while for the case of 1.88 eV (plasmon) excitation, the plexciton reflection spectrum displays barely any optical response (Fig. 2c), similar to the case without pumping (grey dashed lines in Fig. 2). More detailed time-resolved comparisons are provided in Supplementary Note 5. The fact that excitonic excitation can produce the same signal as plexcitonic excitation while plasmonic excitation cannot in our Ag ND–WS_2_ hybrid system firmly suggests that the nonlinearity of plexcitons originates from their excitonic part rather than from the plasmonic part under our experimental conditions. This result is surprisingly coincident with the case of microcavity polaritons, which is reasonable when considering the intrinsically weak optical nonlinearity of pure plasmonic NDs.

**Fig. 3:**
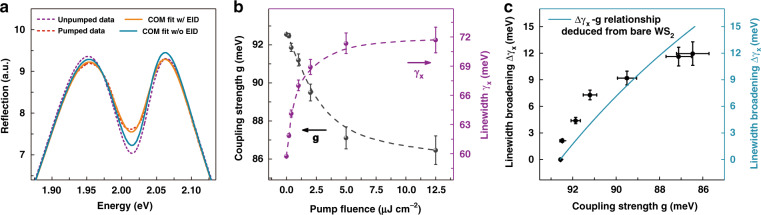
Saturation and dephasing nonlinearities in plexcitons. **a** COM fitting results of the plexciton reflection spectrum at an incident fluence of 2.0 µJ cm^−2^ with (orange solid line) and without (blue solid line) the EID effect. The red dashed line represents the pumped plexciton reflection spectrum at *t* = 100 fs, and the purple dashed line is the plexciton reflection spectrum unaffected by the pump excitation. **b** Plasmon–exciton coupling strength (*g*) and exciton resonance linewidth $(\gamma _{{{\mathrm{x}}}})$ parameters (dots) at various incident fluences obtained from COM fitting. The dashed lines are guides for data classification. **c** Comparison of the ${\Delta}\gamma _{{{\mathrm{x}}}}$–*g* parameters (black dots) directly extracted from **b** with the ${\Delta}\gamma _{{{\mathrm{x}}}}$–*g* relationship (blue solid line) indirectly derived from the bare WS_2_ excitons, in which ${\Delta}\gamma _{{{\mathrm{x}}}}$ denotes the broadening of the exciton resonance linewidth

Given that plexcitons have the same excitonic nonlinearity source as microcavity polaritons, we can analyze their nonlinear interactions similarly to those for microcavity polaritons. Basically, two types of mechanisms govern the nonlinearity of TMD-based microcavity polariton devices^21–26^: (i) the repulsive Coulombic exchange interaction ($\beta _{{{\mathrm{x}}}}$) between excitons, which normally yields a blueshift of the resonance energy, and (ii) the phase space filling (i.e., Pauli blocking)-induced excitonic fermionic saturation interaction ($\beta _{{{\mathrm{s}}}}$), which often reduces the exciton-photon coupling strength. Thus, *β*_x_ and *β*_s_ are also exploited here to explain the nonlinear behaviours of plexciton reflection spectra. However, by only accounting for the *β*_x_ and *β*_s_ nonlinearities i.e., the variation in the exciton resonance energy (*E*_x_) and plasmon–exciton coupling strength (*g*) in COM analysis, we cannot reproduce the measured plexciton reflection spectral curves at different incident fluences, as shown in Fig. 3a and Fig. S11 (blue solid lines), especially the linewidth broadening features of the plexciton resonance. A higher-order Coulomb correlation effect, i.e., the excitation-induced dephasing (EID), has been recently found to play a nonnegligible role in TMDs with increasing exciton density^53,54^, which typically gives rise to broadening of the exciton resonance linewidth (${\upgamma}_{{{\mathrm{x}}}}$). As a result, we assume that an extra nonlinearity induced by EID^55,56^, called the dephasing nonlinearity ($\beta _{{{\mathrm{d}}}}$) here, should also contribute to the whole plexciton nonlinearity in addition to the exchange ($\beta _{{{\mathrm{x}}}}$) and saturation ($\beta _{{{\mathrm{s}}}}$) nonlinearities since plexcitons are naturally localized in their spatial distribution and the close interparticle distance between plexcitons will facilitates the appearance of higher-order many-body effects. With the aid of EID, i.e., the change in the exciton resonance linewidth (*γ*_x_) in COM analysis, the measured plexciton reflection spectral curves can be well reproduced (orange solid lines in Fig. 3a and Fig. S11). Correspondingly, the COM fitting parameters obtained from the measured plexciton reflection spectra as a function of incident fluence are selectively plotted in Fig. 3b (only *g* and ${\upgamma}_{{{\mathrm{x}}}}$ are displayed) and fully listed in Table S2, which numerically demonstrate the existence of $\beta _{{{\mathrm{x}}}}$, $\beta _{{{\mathrm{s}}}}$, and $\beta _{{{\mathrm{d}}}}$ nonlinearities in our plexcitonic system (i.e., power-dependent monotonic blueshift of the exciton energy, reduction of the plasmon–exciton coupling strength, and linewidth broadening of the exciton resonance).

To experimentally prove the accuracy of the above fitting results, we carefully compare the ${{{\mathrm{{\Delta}}}\upgamma }}_{{{\mathrm{x}}}}$–*g* data directly extracted from the Ag ND–WS_2_ plexcitons (black dots) with the ${{{\mathrm{{\Delta}}}\upgamma }}_{{{\mathrm{x}}}}$–*g* relationship indirectly deduced from the bare WS_2_ excitons (blue solid line) in Fig. 3c (see Supplementary Note 7 for the exhaustive derivation process of ${{{\mathrm{{\Delta}}}\upgamma }}_{{{\mathrm{x}}}}$–*g* relationship from the pump–probe experiments of bare WS_2_ sample). Because the nonlinearity of plexcitons in our study merely comes from their excitonic component, we expect the ${{{\mathrm{{\Delta}}}\upgamma }}_{{{\mathrm{x}}}}$–*g* data from the Ag ND–WS_2_ plexciton system to obey a change trend identical to the ${{{\mathrm{{\Delta}}}\upgamma }}_{{{\mathrm{x}}}}$–*g* relationship from the bare WS_2_ exciton system. The fact that the ${{{\mathrm{{\Delta}}}\upgamma }}_{{{\mathrm{x}}}}$–*g* relationships of plexcitons and excitons approximately overlap in Fig. 3c confirms the validity of our fitting results and further verifies the rationality of our assumption that TMD-based plexcitons have an extra EID-induced nonlinearity in addition to the commonly reported exchange and saturation nonlinearities in TMD-based microcavity polaritons. Notably, the slight deviation of the two ${{{\mathrm{{\Delta}}}\upgamma }}_{{{\mathrm{x}}}}$–*g* relationships at the beginning fluences is potentially due to the plasmon-mediated enhanced exciton-exciton interaction in our plexcitonic system. Nevertheless, to fully interpret the deviation, a further dedicated investigation is needed. In our system, the saturation and dephasing nonlinearities dominate the plexciton nonlinearity, as they bring about sizable changes in spectral splitting and resonance linewidth (i.e., line shape). The role of the exchange nonlinearity can be almost omitted, as the produced exciton energy blueshift (<2 meV) is very small relative to the linewidth of plexcitons (>300 meV), which makes inducing a substantial change in the spectrum difficult. This statement is consistent with the recent literature declaring that the exchange interaction is the dominant nonlinear mechanism at low and intermediate exciton densities (long interparticle distance), while saturation and higher-order interactions govern the polariton nonlinearity at high exciton densities (short interparticle distance)^23,25,57^. Overall, it is the nanoscale confinement of plexcitonic mode inherited from the subwavelength mode volume of plasmonic mode that makes the generated plexcitons tend to be spatially dense^32^, which is why the saturation and dephasing interaction innately predominate the plexciton nonlinearity in our work. Besides, the energy potential traps induced funnelling effects are unlikely to be the source responsible for the confinement of the ML WS_2_ excitations here as they might transform 2D band-like excitons into quasi-0D band-like excitons and thus leading to a suppression of EID effect^26^. Unfortunately, the effective interaction constant of plexciton nonlinearity is hard to quantitatively determine, as we cannot precisely estimate the excited plexciton density due to the nonuniform localized distribution. However, thinking differently, we care more about the pulse energy density used to produce the nonlinearity instead of the excited plexciton density in real life. Compared with the bare WS_2_ exciton system, the pulse energy density required to produce the same magnitude of nonlinear response signals (i.e., oscillator strength bleaching, and resonance linewidth broadening) in the Ag ND–WS_2_ plexciton system is almost 10 times smaller (see Supplementary Note 8 for a detailed evaluation of plexcitons nonlinearity in our work). This undoubtedly corroborates the giant nonlinear interactions between plexcitons.

Ultimately, to guarantee the universality of the above conclusion, we further investigated the plexciton nonlinearity at increased plasmon–exciton coupling strengths (achieved by varying the layer number of WS_2_), as presented in Supplementary Note 9. Pump–probe experiments indicate that the saturation and dephasing nonlinearities still dominate the plexciton nonlinearity under these distinct coupling strengths. However, the stricter criterion for entering strong coupling regime (${{{{g}}}} \,>\, (\gamma _{{{\mathrm{p}}}} + \gamma _{{{\mathrm{x}}}})/2$) is not yet realized here, in which the mechanism of plexciton nonlinearity might become different, especially for the so-called ultrastrong coupling regime^58^. Overall, the plexciton nonlinearity in our work is more akin to nonlinear Fano interference-type behaviour than to Rabi splitting-type behaviour^59–61^, which is only appliable for the intermediate coupling regime and the very onset of the strong coupling regime.

### Tuneable plexcitons for designed ultrafast optical nonlinearity

**Fig. 4:**
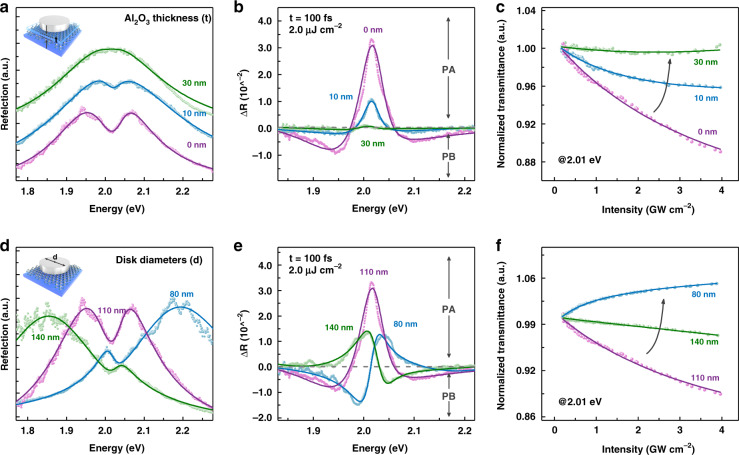
Tuning plexcitons for controllable ultrafast NLA. **a** Reflection spectra, **b** differential reflection spectra, and (**c**) NLA experimental results of Ag ND–WS_2_ samples with different Al_2_O_3_ thicknesses. The dielectric interlayer Al_2_O_3_ was deposited on top of the ML WS_2_ via the atomic layer deposition technique. **d** Reflection spectra, **e** differential reflection spectra, and **f** NLA experimental results of Ag ND–WS_2_ samples with distinct disk diameters. The NLA experimental results of the bare substrate, Ag NDs, and WS_2_ were also measured and are presented in Fig. S22, providing a consistency check of our NLA experiments

The inherently giant nonlinear interaction in plexcitons provides us with an exciting opportunity to tailor the third-order optical nonlinearity of the solid-state system at low excitation energy^60,62^. Particularly, the saturation and dephasing nonlinearities, which can both result in obvious changes in the SW in plexcitons, are very promising for designing the ultrafast nonlinear absorption (NLA) property of the system at the resonance energy when considering the great flexibility of plexcitonic devices. In this section, we demonstrate tuning of the ultrafast NLA property in plexcitons by simply adjusting the structural parameters. Figure 4a shows the reflection spectra of Ag ND–WS_2_ samples with different dielectric interlayer thicknesses. The spectral dip (i.e., SW) in the reflection decreases for thicker alumina (Al_2_O_3_) interlayers since the coupling strength decreases as the field overlap between plasmons and excitons is reduced^63^. Figure 4b further records the differential reflection (${\Delta}{{{{R}}}}$) signals of these Ag ND–WS_2_ samples at *t* = 100 fs under 2.0 µJ cm^−2^ resonant excitation (complete reflection spectral signals can be accessed in Supplementary Note 10), where stronger positive photoinduced absorption (PA) and negative photoinduced bleaching (PB) signals are observed for thinner Al_2_O_3_ interlayers. Based on this dielectric-thickness-dependent nonlinear optical response feature, we developed plexcitonic devices with magnitude-tuneable reverse saturable absorption (RSA) responses at the dip energy position (i.e., 2.01 eV), as exhibited in Fig. 4c. Here, the NLA behaviours of plexcitons were measured by a micro-I-scan technique in transmission geometry (see “Methods”). This result is obviously reasonable, as PA signals at the dip energy position are originally anticipated to produce RSA responses (a PA/PB signal means that more/less probe light is absorbed after pump excitation). Similarly, another degree of freedom of plexcitons, i.e., the diameter of Ag NDs, can also be exploited to tune the NLA property of the system. Figure 4d displays the reflection spectra of Ag ND–WS_2_ samples with distinct disk diameters (i.e., plasmon resonance energies). The dip position substantially shifts with the plasmon resonance energy due to the coherent constructive enhancement and destructive suppression effect in Fano interference. The corresponding positions of the PA and PB signals also vary in the ${\Delta}{{{{R}}}}$ spectra, as shown in Fig. 4e and Fig. S21. For the same detection energy (i.e., 2.01 eV), positive PA signals emerge in destructive interference cases (110 nm, 140 nm), while negative PB signals appear in constructive interference situations (80 nm). Taking advantage of this, the NLA behaviour of plexcitons can be tuned from an RSA response to a saturable absorption (SA) response, as presented in Fig. 4f. The arbitrarily adjustable ultrafast NLA property reveals that plexcitons are a highly promising platform for nonlinear optics applications, which can meet the fundamental requirements for future low-energy ultrafast optical modulation^64^ and all-optical neuromorphic computing^65^.

## Discussion

Recently, we noted that the plasmon-induced hot-electron transfer (PHET) mechanism also plays a significant role in plexcitonic systems^66,67^. To determine whether PHET has an impact on the measured plexciton nonlinearity in our work, we compared the ultrafast responses of two Ag ND–WS_2_ samples with and without a 2 nm Al_2_O_3_ interlayer, which was used to block the potential PHET process. The nearly identical optical signals and absence of a bandgap renormalization effect (originating from coherent doping of plasmonic hot electrons) in both cases in Fig. S23 suggest that PHET is not a prominent process in our results. Moreover, the possible influences from the valley-selective resonant optical Stark effect in TMD-based plexcitons can also be eliminated, as the pump and probe beams are cross-circularly polarized^68,69^.

In summary, the observed giant nonlinearity in plexcitons is mainly dominated by the saturation and dephasing interactions from their excitonic component. Together with their great tunability and small footprint, plexcitonic devices serve as an outstanding platform allowing active design of the ultrafast NLA responses of solid-state systems at small excitation power. Such substantial progress in plexcitons has opened new routes for high-speed all-optical switching and energy-efficient all-optical data processing under room conditions.

## Materials and methods

### Steady-state extinction measurements

The extinction spectra of the samples were measured by an inverted optical microscope system (ARMS, from Ideaoptics Instruments) at room temperature, where a tungsten halogen lamp (100 W) was used as the incident light source to excite the hybrid array at normal incidence. An Olympus objective (MPlanFLN ×100, NA = 0.9) was applied to collect the transmitted light from the sample, and then, the light was directed to a spectrometer equipped with a blazed grating (150 g/mm, blazed at 0.5 μm) and finally analyzed by a thermoelectrical CCD. A two-dimensional slit was placed in the real image plane of the light path to determine the size of the testing area, which was constantly maintained at 20 × 20 µm^2^ throughout our extinction measurements.

### Time-resolved pump–probe measurements

The ultrafast optical responses of the Ag ND–WS_2_ hybrid system were measured by using a home-built time-resolved pump–probe system operating in reflective mode at room temperature. The pump beam, with tuneable excitation energy ranging from 0.77 to 2.61 eV, was produced by pumping a passive optical parametric amplifier (TOPAS) with the output of a Ti:sapphire laser (Spectra-Physics, 1.55 eV, 65 fs, 1 kHz), and its bandwidth was determined by a 10-nm bandpass filter (Thorlabs). A small portion of the Ti:sapphire laser was picked off to drive a sapphire crystal, which generated supercontinuum white light that spanned energies from 1.13 to 2.75 eV and served as the probe beam. The time delay between the pump and probe beam was precisely controlled by using a mechanical delay line in the optical path of the probe beam. The pair of fs laser pulses was then sent together to the surface of the sample by a long-focus objective (Olympus, SLMPLN 20×, NA = 0.25) with a focused spot diameter of 12 μm (pump) and 5 μm (probe). Finally, the reflected probe pulses were collected and analyzed by a fibre-coupled multichannel spectrometer, and the energy-dependent chirp of the white light probe pulse was beforehand corrected by measuring the ultrafast response of a bare thin gold foil. In this work, we used probe reflection ${{{{R}}}}({{{{t}}}}) = {{{{R}}}}_{{{{\mathrm{Ag}}}}\;{{{\mathrm{ND}}}} - {{{\mathrm{WS}}}}_2}({{{{t}}}})/{{{{R}}}}_{{{{\mathrm{quartz}}}}}$ as the main recorded form of observations, where ${{{{R}}}}_{{{{\mathrm{Ag}}}}\;{{{\mathrm{ND}}}} - {{{\mathrm{WS}}}}_2}({{{{t}}}})$ and ${{{{R}}}}_{{{{\mathrm{quartz}}}}}$ denote the reflected light of the Ag ND–WS_2_ hybrid system (with or without pump excitation) and bare quartz, respectively. Correspondingly, the differential form of the probe reflection signal can also be acquired as ${\Delta}{{{{R}}}}\left( {{{{t}}}} \right) = \log _{10}({{{{R}}}}({{{{t}}}})_{{{{\mathrm{pump}}}}\;{{{\mathrm{on}}}}}/{{{{R}}}}_{{{{\mathrm{pump}}}}\;{{{\mathrm{off}}}}})$, where ${{{{R}}}}({{{{t}}}})_{{{{\mathrm{pump}}}}\;{{{\mathrm{on}}}}}$ represents the probe reflection perturbed by pump excitation at a selected time delay and ${{{{R}}}}_{{{{\mathrm{pump}}}}\;{{{\mathrm{off}}}}}$ is the probe reflection unaffected by pump excitation. Both forms of the probe reflection signal were utilized to study the experimental results in this text depending on the specific discussion. To ensure a good signal-to-noise ratio, the signal was obtained by averaging the data from 45k spectra, and the influence of laser intensity fluctuations was eliminated by introducing a reference beam. Importantly, the zero time delay (*t* = 0 fs) in our study is defined as the moment when the probe pulse impinges on the sample concurrently with the tailing edge of the pump pulse (i.e., the time when transient grating signals almost disappear).

Note that an unconventional pump–probe detection setup was adopted in our measurements to achieve completely resonant pump excitation of plexcitons (i.e., the pump energy is spectrally degenerate with the excitonic and plasmonic resonance energies). Specifically, the pump beam was set to oblique incidence with respect to the probe beam under the microscope, which ensured efficient spatial separation between the pump and the probe path. The spatial isolation between the pump and probe pulses allows high-quality extraction of the probe signal from the stray pump signal background. However, this method still cannot exclude all the stray pump signals, as pump pulses will be scattered into the probe path by the Ag NDs. Therefore, a polarization isolation configuration was further employed in the pump–probe system to suppress the excess scattered pump signal. Specifically, the polarizations of the pump and probe pulses were adjusted to be cross-circularly polarized before reaching the sample. Using a quarter-wave plate and a linear polarizer in front of the collecting fibre port, the reflected cross-circularly polarized pump and probe pulses were converted into two orthogonal linearly polarized pulses, which were then selectively filtered by a linear polarizer, thereby inhibiting unwanted pump scattering. The application of the spatial and polarization isolation detection setup in our pump–probe system guarantees a high degree of extinction of the stray pump signal. This enables completely resonant pump excitation measurements of plexcitons to be performed.

### Nonlinear transmission measurements

To explore the NLA properties of Ag ND–WS_2_ hybrid samples, a home-built open-aperture micro-I-scan system was employed. The photon energy of the excitation pulse was fixed at 2.01 eV in our I-scan measurements, which was generated from an optical parametric amplifier (TOPAS) with the output of a Ti:sapphire laser (Spectra-Physics, 1.55 eV, 65 fs, 1 kHz). A long working distance objective (Mitutoyo, M Plan Apo NIR, 50×, NA = 0.42) was applied to focus the incident light onto the sample with a spot diameter of ~ 4 µm. A continuously adjustable neutral density filter (Thorlabs, NDL-10C-4) was adopted to vary the laser intensity, and a chopper (Thorlabs, MC2000B-EC) was utilized to modulate the laser frequency from 1 kHz to 500 Hz. Finally, two Si detectors with dual-channel lock-in amplifiers (Sine Scientific Instrument, OE1022D) were used to measure the transmitted and incident light intensities.

### Theorical modelling

To extract the key parameters (i.e., peak energies and linewidths of the plasmon and exciton resonance, as well as the plasmon–exciton coupling strength) from the measured reflection spectra of the Ag ND–WS_2_ plexcitonic system, an intuitive COM was developed here to analyze the experimental data, in which both plasmons in the Ag NDs and excitons in the ML WS_2_ were treated as two classical dissipative harmonic oscillators coupled to each other with a strength ${{{\mathrm{g}}}}$. Solving the equations of motion in the COM (see Supplementary Note 2.1 for more details), the extinction cross-section (i.e., dissipation of energy as a function of frequency) of the combined system with two coupled resonances can be stated as^41^

1 $$\sigma \left( {{{{E}}}} \right) \propto {{{\mathrm{EIm}}}}\left\{ {\frac{{({{{{E}}}}_{{{\mathrm{x}}}}^2 - {{{{E}}}}^2 + {{{\mathrm{i}}}}\gamma _{{{\mathrm{x}}}}{{{{E}}}})}}{{({{{{E}}}}_{{{\mathrm{p}}}}^2 - {{{{E}}}}^2 + {{{\mathrm{i}}}}\gamma _{{{\mathrm{p}}}}{{{{E}}}})({{{{E}}}}_{{{\mathrm{x}}}}^2 - {{{{E}}}}^2 + {{{\mathrm{i}}}}\gamma _{{{\mathrm{x}}}}{{{{E}}}}) - {{{{E}}}}^2{{{{g}}}}^2{{{{e}}}}^{ - 2{{{\mathrm{i}}}}\left( \varphi \right)}}}} \right\}$$

where ${{{{E}}}}_{{{\mathrm{x}}}}$, ${{{{E}}}}_{{{\mathrm{p}}}}$, $\gamma _{{{\mathrm{x}}}}$, and $\gamma _{{{\mathrm{p}}}}$ represent the peak energy and full width at half maximum (FWHM) of the excitonic and plasmonic resonances, ${{{\mathrm{g}}}}$ is the plasmon–exciton coupling strength (i.e., the energy exchange rate), and $\varphi$ represents the phase shift between the plasmon and exciton resonances. The measured plexciton reflection spectra were fitted using Eq. (1), and the relationship among the fitted *g*, $\gamma _{{{\mathrm{x}}}}$ and $\gamma _{{{\mathrm{p}}}}$ determine the ultimate coupling regime of the whole system. That is, ${{{{g}}}} \ll (\gamma _{{{\mathrm{p}}}} - \gamma _{{{\mathrm{x}}}})/2$ for the weak coupling regime, $(\gamma _{{{\mathrm{p}}}} - \gamma _{{{\mathrm{x}}}})/2 \,<\, {{{{g}}}} \,<\, (\gamma _{{{\mathrm{p}}}} + \gamma _{{{\mathrm{x}}}})/2$ for the intermediate coupling regime, and ${{{{g}}}} \,>\, (\gamma _{{{\mathrm{p}}}} + \gamma _{{{\mathrm{x}}}})/2$ for the strong coupling regime.

Under the resonance coupling condition (i.e., ${{{{E}}}}_{{{\mathrm{p}}}} = {{{{E}}}}_{{{\mathrm{x}}}} = {{{{E}}}}_0$) and assuming $\left| {{{{{E}}}} - {{{{E}}}}_0} \right| \ll {{{{E}}}}$, the peak energy position of the UB and LB plexciton, i.e., ${{{{E}}}}_{{{{\mathrm{UB}}}},{{{\mathrm{LB}}}}}$, can be approximately calculated as ${{{{E}}}}_0 \pm \frac{1}{2}\sqrt {\frac{{\gamma _{{{\mathrm{p}}}} + \gamma _{{{\mathrm{x}}}}}}{{\gamma _{{{\mathrm{p}}}}}}\sqrt {{{{{g}}}}^4 + \gamma _{{{\mathrm{p}}}}\gamma _{{{\mathrm{x}}}}{{{\mathrm{g}}}}^2} - \frac{{\gamma _{{{\mathrm{x}}}}{{{\mathrm{g}}}}^2}}{{\gamma _{{{\mathrm{p}}}}}} - \gamma _{{{\mathrm{x}}}}^2}$. Therefore, the Rabi splitting energy (${{{\mathrm{{\Omega}}}}}$), which is defined as the discrepancy between the ${{{{E}}}}_{{{{\mathrm{UB}}}}}$ and ${{{{E}}}}_{{{{\mathrm{LB}}}}}$ values, can be expressed as

2 $${{{{E}}}}_{{{{\mathrm{UB}}}}} - {{{{E}}}}_{{{{\mathrm{LB}}}}} = {\Omega} = \sqrt {\frac{{\gamma _{{{\mathrm{p}}}} + \gamma _{{{\mathrm{x}}}}}}{{\gamma _{{{\mathrm{p}}}}}}\sqrt {{{{{g}}}}^4 + \gamma _{{{\mathrm{p}}}}\gamma _{{{\mathrm{x}}}}{{{\mathrm{g}}}}^2} - \frac{{\gamma _{{{\mathrm{x}}}}{{{\mathrm{g}}}}^2}}{{\gamma _{{{\mathrm{p}}}}}} - \gamma _{{{\mathrm{x}}}}^2}$$

Furthermore, ${{{{E}}}}_{{{{\mathrm{UB}}}}}$ and ${{{{E}}}}_{{{{\mathrm{LB}}}}}$ are related to ${{{{E}}}}_{{{\mathrm{p}}}}$ and ${{{{E}}}}_{{{\mathrm{x}}}}$ via

3 $${{{{E}}}}_{{{{\mathrm{UB}}}}} + {{{{E}}}}_{{{{\mathrm{LB}}}}} = 2{{{{E}}}}_0 = {{{{E}}}}_{{{\mathrm{p}}}} + {{{{E}}}}_{{{\mathrm{x}}}}$$

The nonlinear responses of the plexciton spectra under different incident fluences were also modelled by Eq. (1), in which the variation in ${{{{E}}}}_{{{\mathrm{x}}}}$ reflects the energy shift of excitons induced by the Coulombic exchange interaction, the variation in ${\upgamma}_{{{\mathrm{x}}}}$ represents the linewidth broadening of excitons caused by EID, and the change in ${{{\mathrm{g}}}}$ denotes the oscillator strength saturation of excitons due to Pauli blocking. The changes in ${{{{E}}}}_{{{\mathrm{p}}}}$ and ${\upgamma}_{{{\mathrm{p}}}}$ of the plasmons can be ignored here because of their innate weak optical nonlinearity compared with the excitons.

## Supplementary information

Supplementary Information for Interacting plexcitons for designed ultrafast optical nonlinearity in a monolayer semiconductor
