# Supplementary material for: Interacting plexcitons for designed ultrafast optical nonlinearity in a monolayer semiconductor
Source: Light Sci Appl. 2022 Apr 14;11:94. doi: 10.1038/s41377-022-00754-3 (PMC9010435; doi:10.1038/s41377-022-00754-3)
Supplement: Supplementary file 1 — Supplementary Information for Interacting plexcitons for designed ultrafast optical nonlinearity in a monolayer semiconductor [file 41377_2022_754_MOESM1_ESM.docx]

Supplementary Information for Interacting plexcitons for designed ultrafast optical nonlinearity in a monolayer semiconductor

Yuxiang Tang^1,†^, Yanbin Zhang^2,†^, Qirui Liu^1^, Ke Wei^1,2,4^, Xiang’ai Cheng^1^, Lei Shi^2,*^,

Tian Jiang^1,4,*^

^*^Corresponding author: [tjiang@nudt.edu.cn](mailto:tjiang@nudt.edu.cn)

^*^Corresponding author: lshi@fudan.edu.cn

^1^College of Advanced Interdisciplinary Studies, National University of Defense Technology, Changsha 410073, China.

^2^Key Laboratory of Micro- and Nano-Photonic Structures (Ministry of Education), and State Key Laboratory of Surface Physics, Department of Physics, Fudan University, Shanghai 200433, China
^3^State Key Laboratory of High Performance Computing, College of Computer, National University of Defense Technology, Changsha, 410073, China.
^4^Beijing Institute for Advanced Study, National University of Defense Technology, Beijing 100000, China.

^†^These authors contribute equally

**Supplementary Note 1: Sample preparation and characterization**

- 1. **Fabrication details and sample morphology**

To fabricate the hybrid Ag ND-WS_2_ hybrid devices, precleaned fused silica substrates were firstly prepared. Subsequently, thin films of monolayer WS_2_ with a typical fake side length of 50-70 µm, synthesized by chemical vapor deposition (CVD) methods, were transferred onto these fused silica substrates using wet transfer technique. Nanodisk arrays structures (100 µm × 100 µm) were then defined and patterned directly on the top of monolayer WS_2_ flakes by electron beam lithography. Finally, 30 nm thick silver was deposited via electron-beam evaporation followed by a lift-off process. All samples in the study were encapsulated by a 200-nm-thick layer of polymethyl methacrylate (950 PMMA A4, spin coating at 4000 r.p.m. for 40 s) to prevent degradation during testing.

Figure S1a, b shows the optical microscopy and scanning electron microscopy images of Ag ND-WS_2_ hybrid system at zero detuning condition. The array period is observed to be around 300 nm and the diameter of nanodisk is nearly 110 nm. In addition, a typical transmission spectrum of individual monolayer WS_2_ flake is characterized in Figure S1c. The intense and sharp exciton peak at 2.0 eV indicates the good quality of our sample.


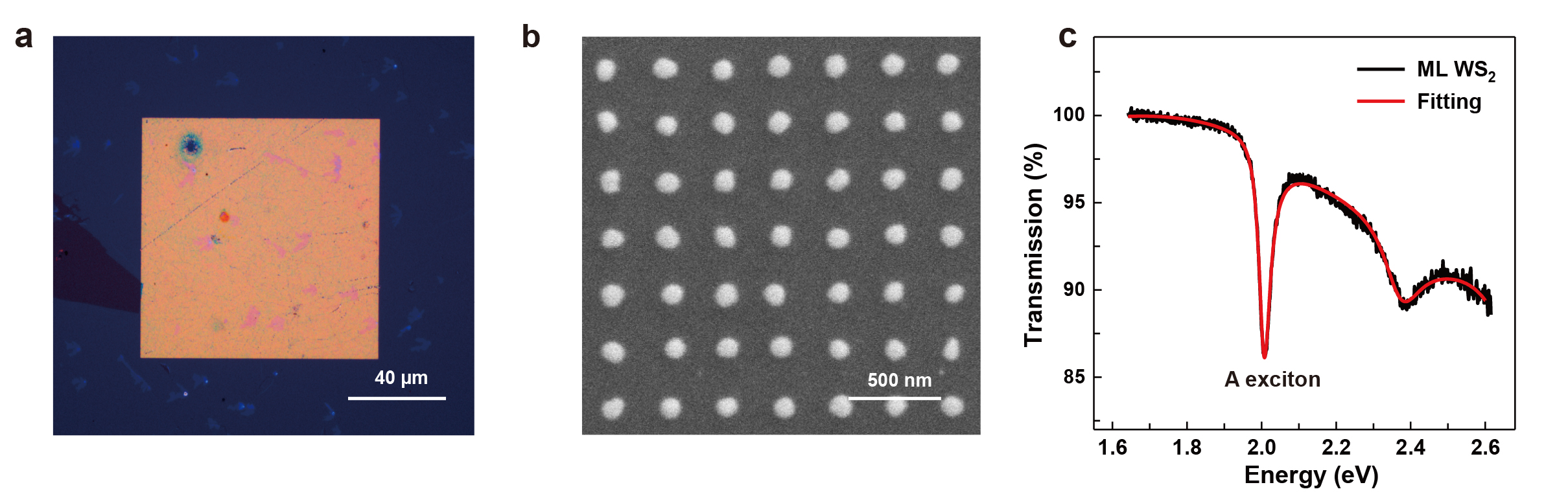


Figure S1. (a) Optical microscopy and (b) Scanning electron microscopy images of Ag ND-WS_2_ hybrid structure. (c) Transmission spectrum of the WS_2_ monolayer semiconductor.

- 1. **The influences of diffractive modes in metallic arrays**

In silver nanodisk arrays, the responses of localized surface plasmon resonance provided by single nanodisk are not only dependent on its structural parameters (e.g., disk diameter) but also significantly influenced by its neighbouring nanodisks via near-flied or far-field electromagnetic coupling^1-3^. Based on research aim of our work (exploiting the nonlinearity of naturally localized plexcitons), neither near-filed nor far-field interaction between silver nanodisks is wanted in our work since their existence may change localized nature of original plasmonic modes. For the near-filed coupling effect (plasmon-plasmon interaction), its influence can be firstly excluded in our structure as it works only when nanodisks are relatively densely packed (our lattice period is comparably too large to achieve efficient near-field interaction). For the far-filed coupling effect (plasmon-lattice interaction), its appearance is strongly dependent on the energy dispersion relationship between diffractive modes (provided by lattice period) and plasmonic modes (provided by single nanodisk) of the structure. When the resonance energy of diffractive modes gets close to that of plasmonic modes, the far-field coupling between nanodisks becomes significant and the localized plasmonic modes will take on the diffractive character of the photonic lattice modes.

Therefore, the period of lattice in our work is carefully chosen to be 300 nm since the diffractive modes are sufficiently separated from plasmonic modes at this lattice period, as shown in the angle-resolved transmission spectrum of Figure S2a. For comparison, the angle-resolved transmission spectrum of a 350-nm-period sample (other structure parameters keep unchanged) is also presented in Figure S2b. Here, the diffractive modes of the lattice show an obvious redshift and strongly interact with plasmonic modes, which consequently results in an observable bending of the plasmonic modes. This phenomenon reveals that the far-flied coupling effect gradually appears at increasing lattice period and the proper selection of lattice period is necessary for our structure.


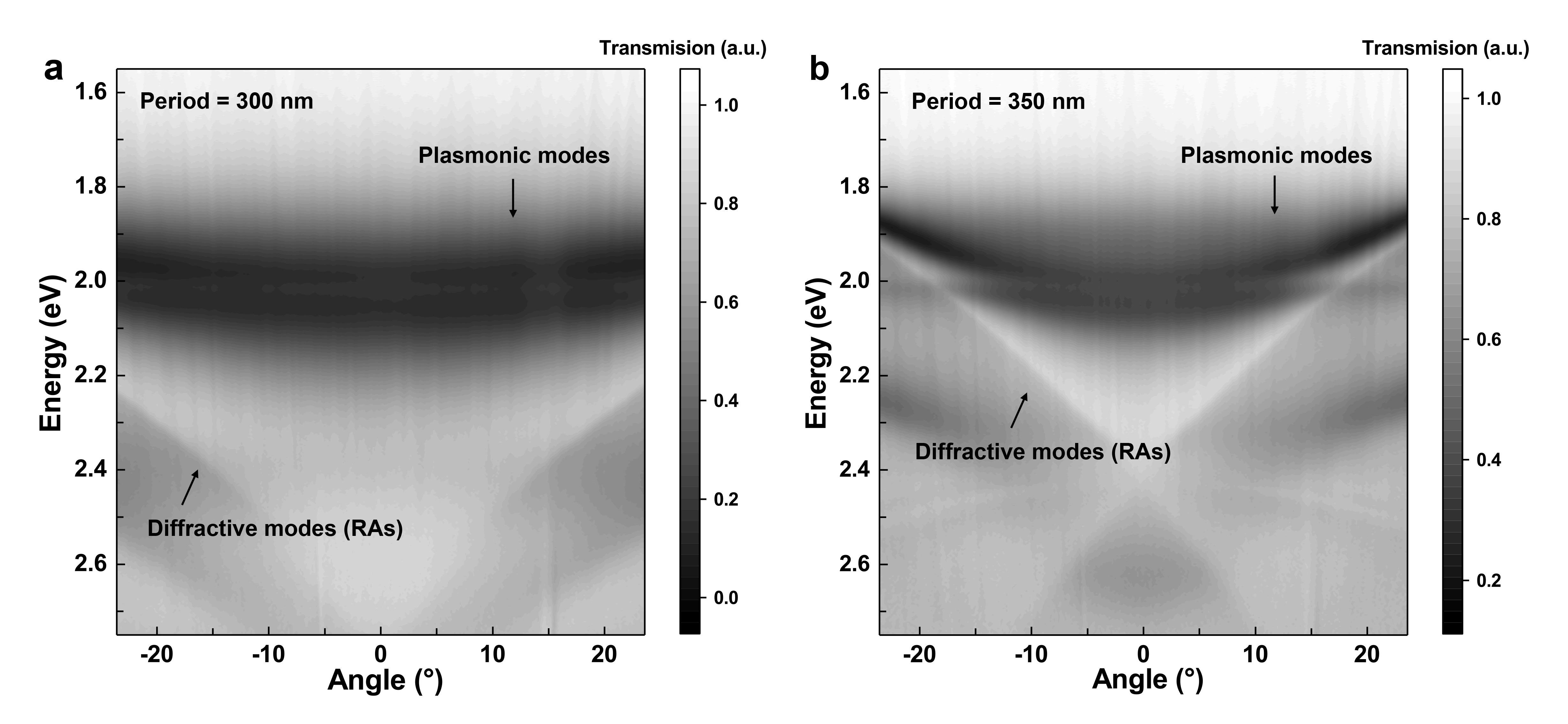
.

Figure S2. Angle-resolved transmission spectrum of Ag ND-WS_2_ hybrid device at (a) 300 nm and (b) 350 nm period with the same disk diameter. An evident redshift of diffractive modes can be clearly seen at a larger lattice period (350 nm), which leads to a change of the plasmonic modes.

**Supplementary Note 2: Coupled oscillator model and fitting results**

- 1. **Derivation of Coupled oscillator model**

The classical coupled oscillator model theory has been widely used to describe the Fano interference or strong coupling interaction between two quantum systems^4-6^. In this approach, the system is modeled as two dissipative harmonic oscillators with a coupling spring connecting with each other. For the hybrid plasmon-exciton system in our work, it can be initially assumed that only the LSPR of Ag ND is predominately excited by the external field since the extinction cross-section of the LSPR is much larger than the exciton resonance (the exciton of WS_2_ is subsequently driven through it coupling to the LSPR). Thus, the motional equations of the coupled plasmon-exciton system can be described as:

$$\begin{aligned} \ddot{x}_{\mathrm{pl}}+\gamma_{\mathrm{pl}}\dot{x}_{\mathrm{pl}}+\omega_{\mathrm{pl}}^{2}x_{\mathrm{pl}}+g\dot{x}_{\mathrm{ex}}=F_{\mathrm{pl}}e^{-i\left( \omega t \right)} \#\left( S1 \right) \end{aligned}$$

$\begin{aligned} \ddot{x}_{\mathrm{ex}}+\gamma_{\mathrm{ex}}\dot{x}_{\mathrm{ex}}+\omega_{\mathrm{ex}}^{2}x_{\mathrm{ex}}-g\dot{x}_{\mathrm{pl}}=0 \#\left( S2 \right) \end{aligned}$

where $x_{\mathrm{pl}}$ and $x_{\mathrm{ex}}$ stand for the displacements of the plasmonic and excitonic oscillators, respectively*,* $\omega_{\mathrm{pl}}$, $\omega_{\mathrm{ex}}$ and $\gamma_{\mathrm{pl}}$, $\gamma_{\mathrm{ex}}$ are the resonant frequency and full width at half maximum (FWHM) of the excitonic and the plasmonic resonance, respectively, $g$ is the plasmon-exciton coupling strength and $F_{\mathrm{pl}}(t)$ represents the driving electric field. In fact, the assumption that all excitons are driven by plasmon is not very accurate, especially under the circumstances that the oscillator strength of excitons is not much smaller than the oscillator strength of plasmons or the incident excitation fluence is large enough to produce excitons directly. Thus, A phase difference between $\theta$ and $\phi$ should be considered in the displacement of plasmonic ($\theta$) and excitonic resonance ($\phi$)^7-9^, and the resulting phase shift ($\varphi=\theta-\phi$) between plasmon and exciton should also be accounted into the complex coupling constant $\tilde{g}$, which are stated as followed:

$$\begin{aligned} x_{\mathrm{pl}}\left( t \right)=e^{-i\theta}x_{\mathrm{pl}}\left( \omega\right)e^{-i\left( \omega t \right)} \#\left( S3 \right) \end{aligned}$$

$$\begin{aligned} x_{\mathrm{ex}}\left( t \right)=e^{-i\phi}x_{\mathrm{ex}}\left( \omega\right)e^{-i\left( \omega t \right)} \#\left( S4 \right) \end{aligned}$$

$$\begin{aligned} \tilde{g}=ge^{-i\left( \varphi\right)} \#\left( S5 \right) \end{aligned}$$

By solving Equations S1 to S5, we can estimate the extinction cross-section of the coupled system as:

$$\begin{aligned} \sigma_{\mathrm{ex}}\left( \omega\right)\propto\omega Im\left( F_{\mathrm{pl}}x_{\mathrm{pl}}\left( w \right) \right)\propto\omega Im\left\{ \frac{{F_{\mathrm{pl}}(\omega_{\mathrm{ex}}^{2}-\omega}^{2}+i\gamma_{\mathrm{ex}}\omega)}{{(\omega_{\mathrm{pl}}^{2}-\omega}^{2}+i\gamma_{\mathrm{pl}}\omega){(\omega_{\mathrm{ex}}^{2}-\omega}^{2}+i\gamma_{\mathrm{ex}}\omega)-\omega^{2}g^{2}e^{-2i\left( \varphi\right)}} \right\} \#\left( S6 \right) \end{aligned}$$

Considering both scattering light and absorbing light contribute to the reflection of the hybrid structure, the numerical expression of $\sigma_{\mathrm{ex}}\left( \omega\right)$ here can also be used to approximately fit the measured reflection spectrum of the coupled system. The manifestation of spectrum is the result of Fano interference between two resonances with very different linewidths when $g\ll{(\gamma_{\mathrm{pl}}-\gamma_{\mathrm{ex}})}/2$. While for $g\gg{(\gamma_{\mathrm{pl}}-\gamma_{\mathrm{ex}})}/2$, the observed spectrum can be understood as the superposition of two newly-formed normal modes, i.e., mode splitting. Moreover, If the exciton is resonant with the plasmon (i.e., ${\omega_{\mathrm{pl}}\approx\omega_{\mathrm{ex}}\approx\omega}_{0}$), and coupling strength $(g)$, damping rates ($\gamma_{\mathrm{pl}}$, $\gamma_{\mathrm{ex}})$ are small compared to the resonance frequencies $\omega_{0}$, the Equation S6 can be further simplified as (near the resonance peak, $\left| \omega-\omega_{0} \right|\ll\omega$):

$$\begin{aligned} \sigma_{\mathrm{ex}}\left( \omega\right)\approx Im\left\{ \frac{\omega_{0}-\omega-{i\gamma_{\mathrm{ex}}\omega}/2}{{(\omega}_{0}-\omega-{i\gamma_{\mathrm{pl}}\omega}/2)(\omega_{0}-\omega-{i\gamma_{\mathrm{ex}}\omega}/2)-{g^{2}}/4} \right\} \#\left( S7 \right) \end{aligned}$$

where the frequency values of two maximum peak of spectrum $\sigma_{\mathrm{ex}}\left( \omega\right)$ are solved to be $\omega_{\pm}=\omega_{0}\pm\frac{1}{2}\sqrt{\frac{\gamma_{\mathrm{pl}}+\gamma_{\mathrm{ex}}}{\gamma_{\mathrm{pl}}}\sqrt{g^{4}+{\gamma_{\mathrm{pl}}\gamma_{\mathrm{ex}}g}^{2}}-\frac{{\gamma_{\mathrm{ex}}g}^{2}}{\gamma_{\mathrm{pl}}}-{\gamma_{\mathrm{ex}}}^{2}}$. By adding and subtracting these two peak frequencies $\omega_{\pm}$, we can obtain:

$$\begin{aligned} \omega_{+}-\omega_{-}=\Omega=\sqrt{\frac{\gamma_{\mathrm{pl}}+\gamma_{\mathrm{ex}}}{\gamma_{\mathrm{pl}}}\sqrt{g^{4}+{\gamma_{\mathrm{pl}}\gamma_{\mathrm{ex}}g}^{2}}-\frac{{\gamma_{\mathrm{ex}}g}^{2}}{\gamma_{\mathrm{pl}}}-{\gamma_{\mathrm{ex}}}^{2}} \#\left( S8 \right) \end{aligned}$$

$$\begin{aligned} \omega_{+}+\omega_{-}=2\omega_{0}=\omega_{\mathrm{pl}}+\omega_{\mathrm{ex}} \#\left( S9 \right) \end{aligned}$$

Accordingly, the value of $\omega_{+}-\omega_{-}$ (also known as the Rabi splitting, $\Omega$) can be used to characterize the coupling strength between plasmon and exciton. While for $\omega_{+}+\omega_{-}$ , its value can be used to reflect the sum of resonance energy of plasmon and exciton.

- 1. **COM fitting results**

The experimental reflection spectrum of the zero detuning Ag ND-WS_2_ sample ($\omega_{\mathrm{pl}}\approx\omega_{\mathrm{ex}}$) is fitted based on Equation S6 and the relevant results and parameters are presented in Figure S3 and Table S1. In Figure S3, we can see that the fitted curve (red line) matches very well with the experimental reflection spectrum (red dots), showing the good applicability of COM theory in our work. The parameters listed in Table S1 are used to determine the coupling strength (strong, intermediate, or weak) of our hybrid plexcitonic system, as we discussed in the main text.

It should be worth noting here, the experimental spectrum measured by different approaches with different collecting setups are not completely the same even for the same sample location. As shown in Figure S3, the red and black lines represent the reflection spectrum measured via the time-resolved approach and the extinction spectrum measured via the steady-state approach, respectively. Compared with the extinction spectrum, the spectral splitting in reflection spectrum is observed to be much wider and deeper. Besides, the intensity of reflection spectrum is also obviously weaker than the intensity of extinction spectrum at high energy range near the position of WS_2_ B exciton (i.e., from 2.15 eV to 2.3 eV). Comprehensively, the reasons responsible for these spectral differences can be mainly attributed to the following two aspects: (i) The testing area (20 × 20 µm^2^) in the extinction measurements is much larger than the testing area (π × 2.5^^2^ µm^2^) in the reflection measurements. This means the inhomogeneous linewidth broadening effect of LSPR caused by the inhomogeneity of nanodisk ensembles is less introduced in the reflection spectrum, which correspondingly results in a relatively wider and deeper spectral splitting. (ii) the collecting angular range of objective (Olympus, MPlanFLN 100×, NA=0.9) in the extinction measurements is much larger than the collecting angular range (Olympus, SLMPLN 20×, NA=0.25) in the reflection measurements. This means more absorption signals from the WS_2_ constituent of hybrid system are collected in the extinction measurements compared with reflection measurements^10^ (i.e., scattering and absorption signals from the Ag ND constituent of hybrid system, instead of WS_2_ constituent, dominates the reflection spectrum), which leads to the rise of signal intensity at high energy range in the extinction spectrum. Despite the differences, the spectral splitting in both the extinction and reflection measurements undoubtedly indicate the formation of plasmon-exciton hybridization (i.e., intermediate plexcitons) in our sample even if considering the more rigorous criteria for plasmon-exciton interaction under extinction experiments. Reflection spectra are mainly analyzed in the text since all subsequent researches are done based on time-resolved approach.


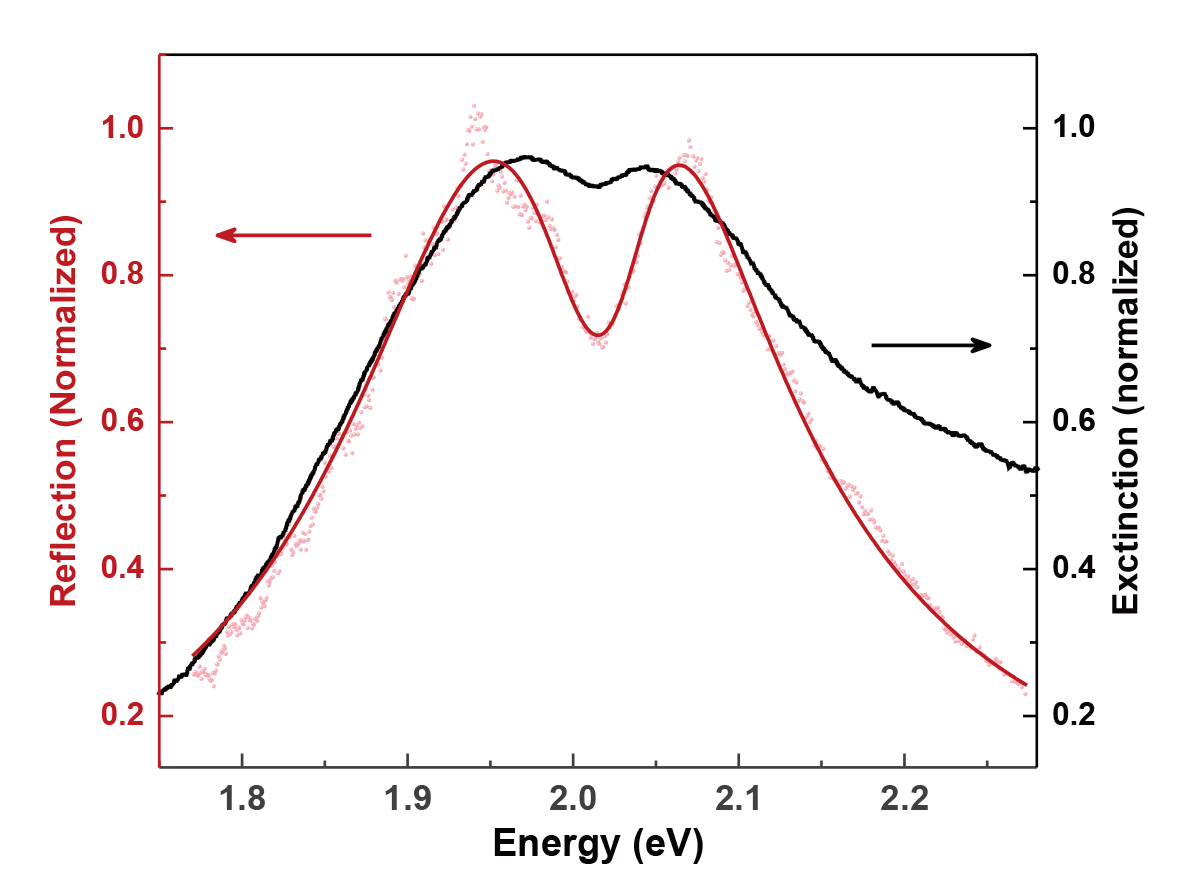


Figure S3. Experimental spectrum of the zero detuning Ag ND-WS_2_ hybrid sample. The red and black lines represent reflection spectrum and extinction spectrum measured via time-resolved approach and steady-state approach, respectively.

|  | $E_{x}$ (eV) | $\gamma_{x}$ (meV) | $E_{p}$ (eV) | $\gamma_{p}$ (meV) | $g$ (meV) | $\varphi$ (π) |
| --- | --- | --- | --- | --- | --- | --- |
| Value | 2.0197 | 59.739 | 1.9966 | 269.85 | 92.564 | -0.0327 |

Table S1. Obtained parameters from the coupled oscillator model fitting in Figure S3 (red line). Here, we replace $\omega_{\mathrm{ex}}$, $\omega_{\mathrm{pl}}$, $\gamma_{\mathrm{ex}}$, $\gamma_{\mathrm{pl}}$ with $E_{x}$, $E_{p}$ ,$\gamma_{x}$, $\gamma_{p}$ in order to be consistent with symbols in the main text.

**Supplementary Note 3:** **Comparison of** **ultrafast dynamics of bare WS_2_, bare Ag ND, and hybrid Ag ND-WS_2_ under resonant excitation**

To clearly distinguish the ultrafast dynamics of coupled system (Ag ND-WS_2_) from the isolated systems (WS_2_, Ag ND) and highlight the different photophysical processes of different excitations (i.e. exciton, plasmon, and plexciton), the time-resolved reflection spectra scans of zero-detuning hybrid Ag ND-WS_2_ plexcitonic system and its individual WS_2_ and Ag ND components are carefully recorded and compared in Figure S4 under resonant pump excitation (2.0 eV) at incident fluences of 5.0 µJ cm^-2^. For the dynamics of bare WS_2_, a sharp single excitonic peak located at 2.014 eV displays evident bleaching and blue-shifting signals after laser pulse excitation, which is normally ascribed to the band filling effect and exchange interaction of excitons. For the dynamics of bare Ag ND, a broad single plasmonic peak centered at 2.021 eV can be observed and shows barely any temporal signals although in the presence of pump excitation, which is due to the inherently weak optical response of pure metallic nanostructures. However, when it comes to the dynamics of the integrated Ag ND-WS_2_ system, a totally different splitting double-peak feature is emerged with lower and higher resonance peaks situated at 1.951 eV and 2.064 eV, respectively. These significant distinctions of dynamics of WS_2_, Ag ND, and Ag ND-WS_2_ systems strongly indicate that the photophysical dynamics of the Ag ND-WS_2_ system is not a simple superposition of the dynamics of isolated WS_2_ and Ag ND, and a valid measure of plexcitons (rather than excitons and plasmons) dynamics in Ag ND-WS_2_ system is achieved in our study.


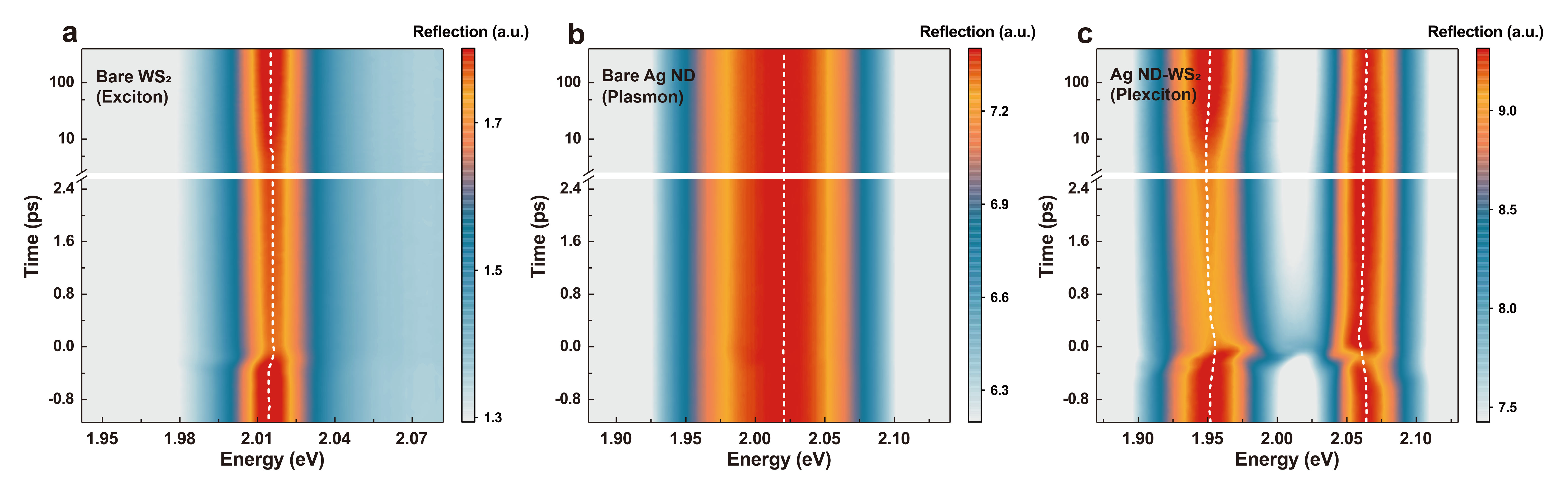


Figure S4. Time-resolved reflection spectra scan of the (a) bare WS_2_ excitonic system, (b) bare Ag ND plasmonic system, and (c) hybrid Ag ND-WS_2_ plexcitonic system under resonant pump excitation (2.0 eV) at incident fluences of 5.0 µJ cm^-2^. The temporal revolutions of peak energy of WS_2_ exciton, Ag-ND plasmon, and Ag ND-WS_2_ plexciton are marked as white dashed lines.

**Supplementary Note 4:** **Ultrafast responses of plexcitons under resonant excitation**

1. **Power-dependent time-resolved reflection spectra scan of Ag ND-WS_2_ plexcitonic system**

Figure S5 demonstrates the power-dependent time-resolved reflection spectra scan of zero-detuning Ag ND-WS_2_ plexcitonic system under resonant pump excitation (2.0 eV). The temporal revolutions of peak energy of UB and LB plexcitons resonances are marked as white dashed lines at every experimental incident fluence.


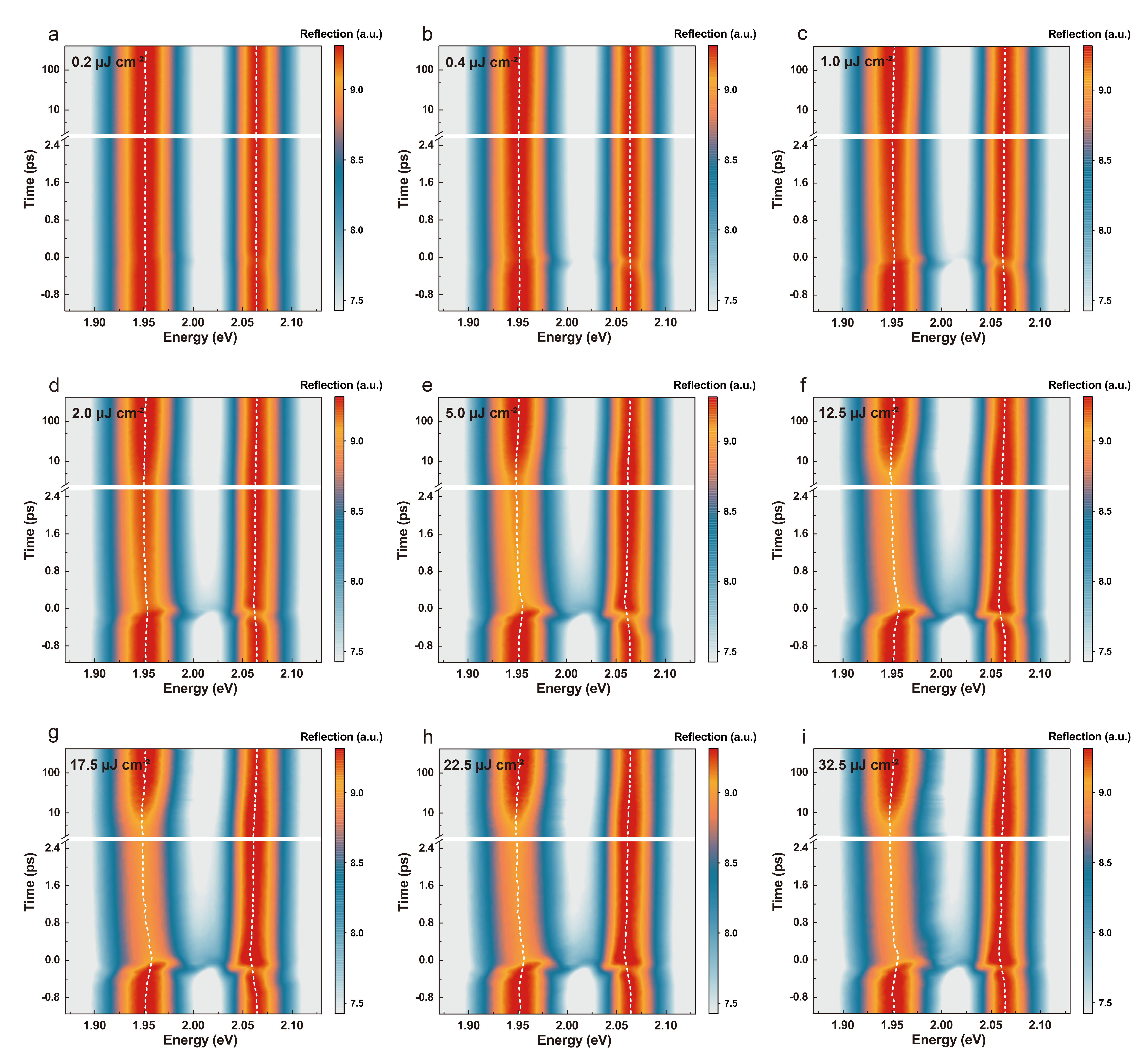


Figure S5. Time-resolved reflection spectra scan of the zero-detuning Ag ND-WS_2_ plexcitonic system under resonant pump excitation (2.0 eV) at incident fluences of (a) 0.2 µJ cm^-2^, (b) 0.4 µJ cm^-2^, (c) 1.0 µJ cm^-2^, (d) 2.0 µJ cm^-2^, (e) 5.0 µJ cm^-2^, (f) 12.5 µJ cm^-2^, (g) 17.5 µJ cm^-2^, (h) 22.5 µJ cm^-2^, (i) 32.5 µJ cm^-2^.

1. **Transient grating effect and peak energy extraction**

As displayed in Figure 1c of the main text, obvious spectral oscillations show up in the time-resolved reflection spectra of our Ag ND-WS_2_ plexcitonic system when the pump and probe pulses approximately overlap with each other (more accurately, the oscillations begin at very early times when probe pulse precedes pump pulse) and this unique feature can be seen more clearly in Figure S6a, b in the form of time-resolved differential reflection spectra (ΔR = log_10_ (R_pump on_/R_pump off_)). These oscillatory structures sustain broadly at the spectral detection range of our experiments (mainly from 1.85 eV to 2.15 eV) and appear only within the very short pump-probe pulses overlapping duration (mainly from -400 fs to 0 fs). Normally, the emergences of transient oscillations in time-resolved differential spectra are primarily derived from three processes: they are (i) pump pulse scattering^11^, (ii) perturbed free induction decay^12-14^, and (iii) transient grating^15,16^, respectively. Here, the effect of pump pulse scattering can be firstly excluded since the bandwidth of pump pulse (filtering by a 10-nm-bandpass filter at 620 nm) is much smaller than the spectral oscillating range and the stray pump pulses scattered into the probe pulse by the Ag NDs are strongly inhibited in our spatially and polarized isolated pump-probe detection configuration. At the same time, the influence from perturbed free induction decay can also be safely ruled out as the appearance of oscillations induced by perturbed free induction decay requires the polarization dephasing time of system is comparable to or longer than the duration of pump pulse, which is unlikely to happen in our coupled plexcitons system. Even for the uncoupled excitons, their existence also cannot produce such broad-range spectral oscillations due to the limited energetic linewidth of exciton despite their dephasing time is comparable to the pump pulse. Therefore, the transient grating effect is most likely responsible for the occurrence of spectral oscillations in our work, especially when the pump beam is incident obliquely with respect to the probe beam. The underneath physical process of transient grating effect can be described as followed: the leading part of pump pulse interferes non-collinearly with the probe pulse at the sample surface, creating a standing wave of laser intensity (i.e., interferometric excitation intensity). The periodic variation of excitation density which is further amplified by LSPR effect greatly modulates the refractive index of sample in a regular spacing across the surface, giving rise to an extraordinary strong diffraction of transient grating (TG) signal filed. Subsequently, the TG signal field is measured by a delayed probe beam and ultimately manifested itself as spectral interference patterns in the frequency domain. It needs to be noted that the TG signal showcased here is a coherent artifact resulted from our oblique incidence pump-probe detection setup and its appearance is almost inevitable because oblique incidence detection setup is necessary for achieving completely resonant pump excitation (i.e., 2.0 eV).

The presence of TG signal superimposed on time-resolved reflection spectra will significantly influence the accurate extraction of peak energy of the UB and LB plexcitons resonance. Thus, eliminating the effect of TG signal is the prerequisite to obtain the right peak energy evolution of E_UB_ and E_LB_ as a function of time, especially for the times when pump pulses overlap with probe pulses. Figure S6c demonstrates the temporal evolution of E_UB_ and E_LB_ with TG signal. It can be clearly seen that the temporal evolution of E_UB_ and E_LB_ is masked by an irregular peak energy change starting nearly from -400 fs until 0 fs, which is attributed to the TG effect. Here, we exclude this abnormal peak energy change by directly replacing it with linearly interpolated data, and the resulted temporal evolution of E_UB_ and E_LB_ without TG signal is shown in Figure S6d.


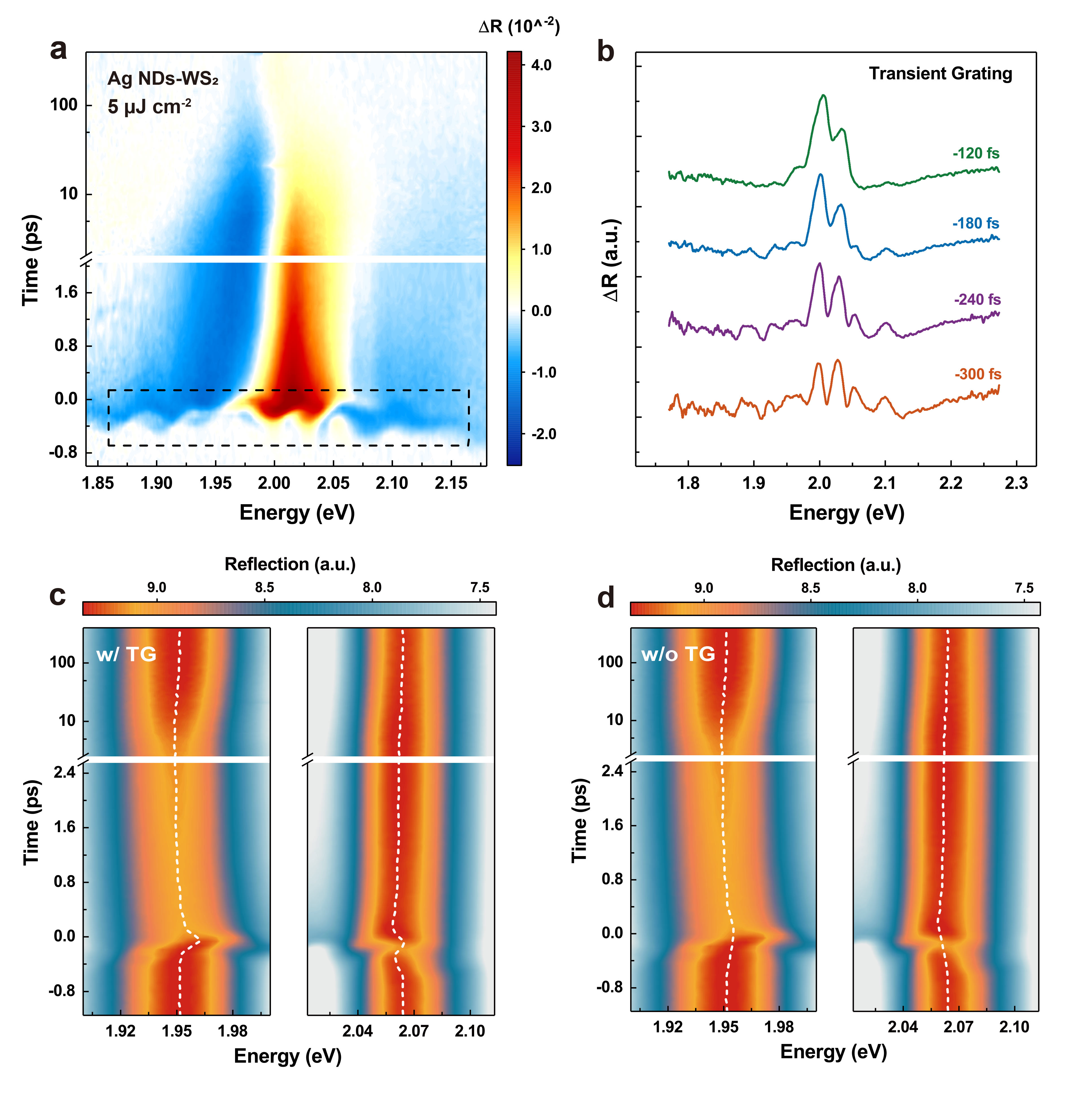


Figure 6. (a) Time-resolved differential reflection spectra of the zero-detuning Ag ND-WS_2_ plexcitonic system under resonant pump excitation (2.0 eV) at incident fluences of 5.0 µJ cm^-2^. The Black dashed box indicates the spectral and temporal range in which oscillations occur. (b) Differential reflection spectra curves at -300fs, -240fs, -180fs, -120fs projected from the cross-section of (a). (c-d) Peak energy evolution of the UB and LB plexcitons resonance as a function of time (c) with TG (d) without TG effect.

1. **Temporal evolution of E_UB_ - E_LB_ and E_UB_ + E_LB_**

In order to precisely observe the differences between the behaviors of UB and LB plexcitons, the peak energy temporal evolution of the E_UB_ and E_LB_ are enlarged in Figure S7a. Correspondingly, the calculated temporal evolution of E_UB_ - E_LB_ and E_UB_ + E_LB_ are presented in Figure S7b, which are utilized in our work to decide the unique occurring time scales of coherent plexcitons, incoherent plasmon/exciton population, and heat effect processes.


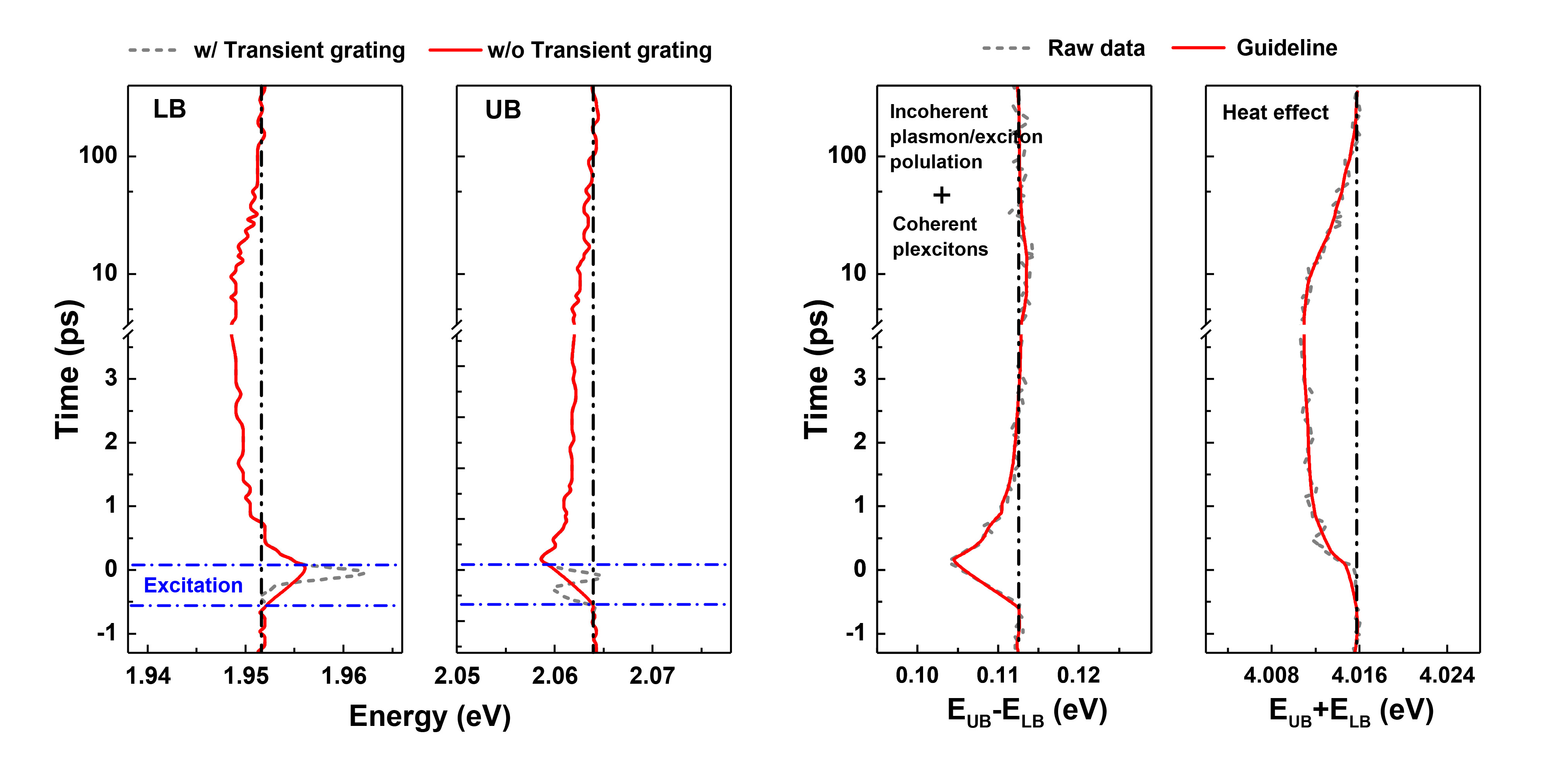


Figure S7. (a) Enlarged temporal evolution of E_UB_ and E_LB_ with/without TG signal. (b) Temporal evolution of E_UB_ - E_LB_ and E_UB_ + E_LB_ acquired by subtracting and adding the E_UB_ and E_LB_ data without transient grating effect (i.e., red lines) in (a).

According to the E_UB_ - E_LB_ and E_UB_ + E_LB_ dynamics in Figure S7b, the ultrafast optical responses of plexcitons after pulsed excitation are well depicted in Figure S8. Specifically, the coherent plexcitons are firstly generated upon excitation and accompanied by the appearance of TG effect. Then, the coherent plexcitons rapidly relax into the incoherent plasmon/exciton population with a typical dephasing time of tens of fs (estimated from the reciprocal of energy splitting value, i.e., 2π/Ω). The subsequent decay (in hundreds of fs) of incoherent plasmon/exciton population results in local heat accumulation in Ag NDs, rising the temperature of adjacent WS_2_ lattice. Finally, the excess heat in the system dissipates out by thermal coupling with the environment at ps timescales.


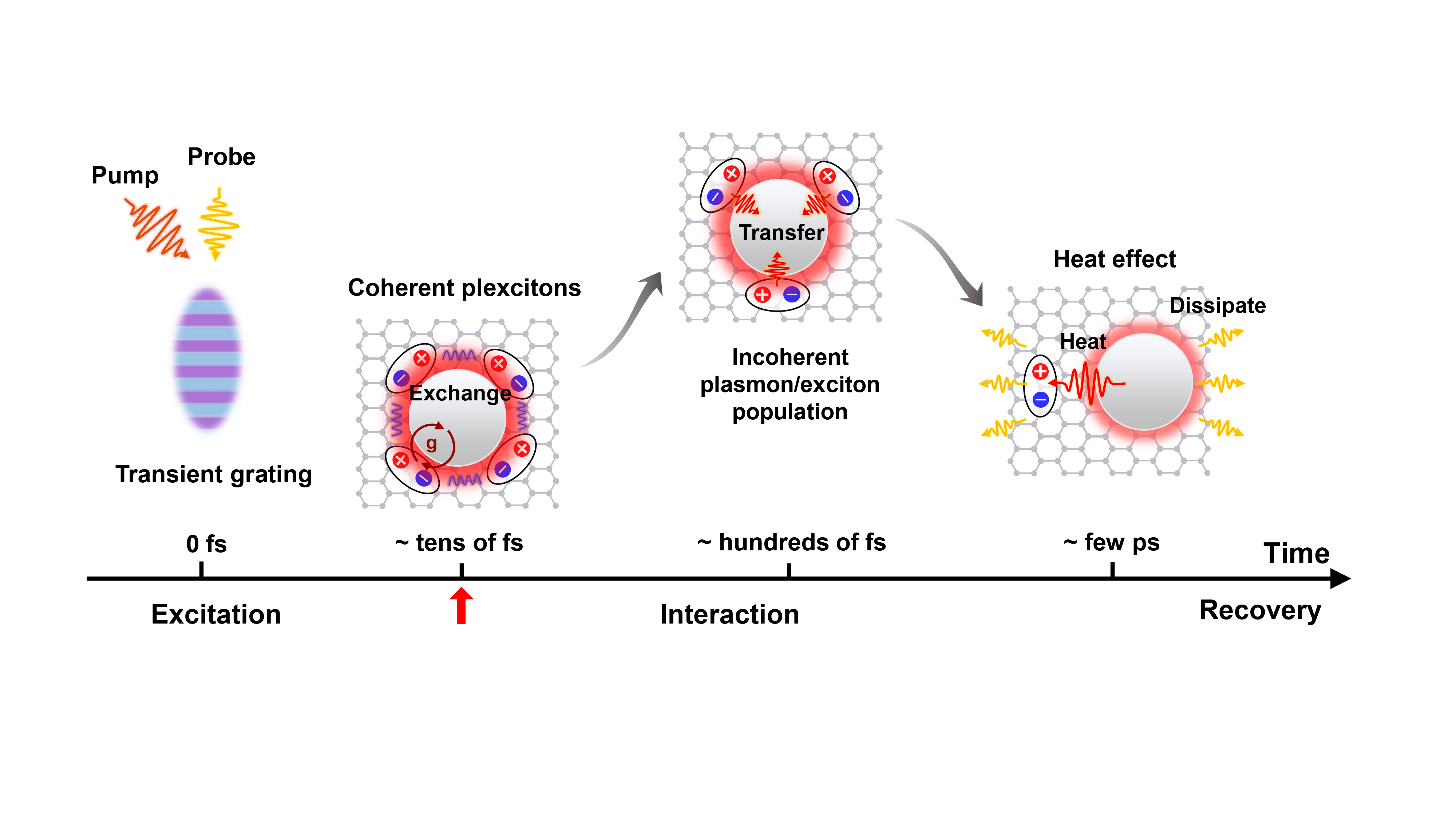


Figure S8. The schematic of plexcitons dynamics after resonant pump pulse excitation.

1. **Transient optical response of bare Ag NDs**

Compared with the ultrafast response of Ag ND-WS_2_ plexcitonic system, the ultrafast response of individual Ag NDs shows barely any signal, as shown in the transient differential reflection spectra of Figure S9. This experimental result indicates that no obvious spectral change (i.e., peak energy shift and linewidth broadening) has happened for LSPR at this pump fluence.

Furthermore, this phenomenon reveals the intrinsically weak optical nonlinearity of pure plasmonic nanoparticles, which can be used as extra evidence supporting the statement that the plexcitons nonlinearity is mainly originated from their excitonic component rather than plasmonic component, as discussed in the ‘Giant nonlinear interaction of plexcitons’ section of the main text.


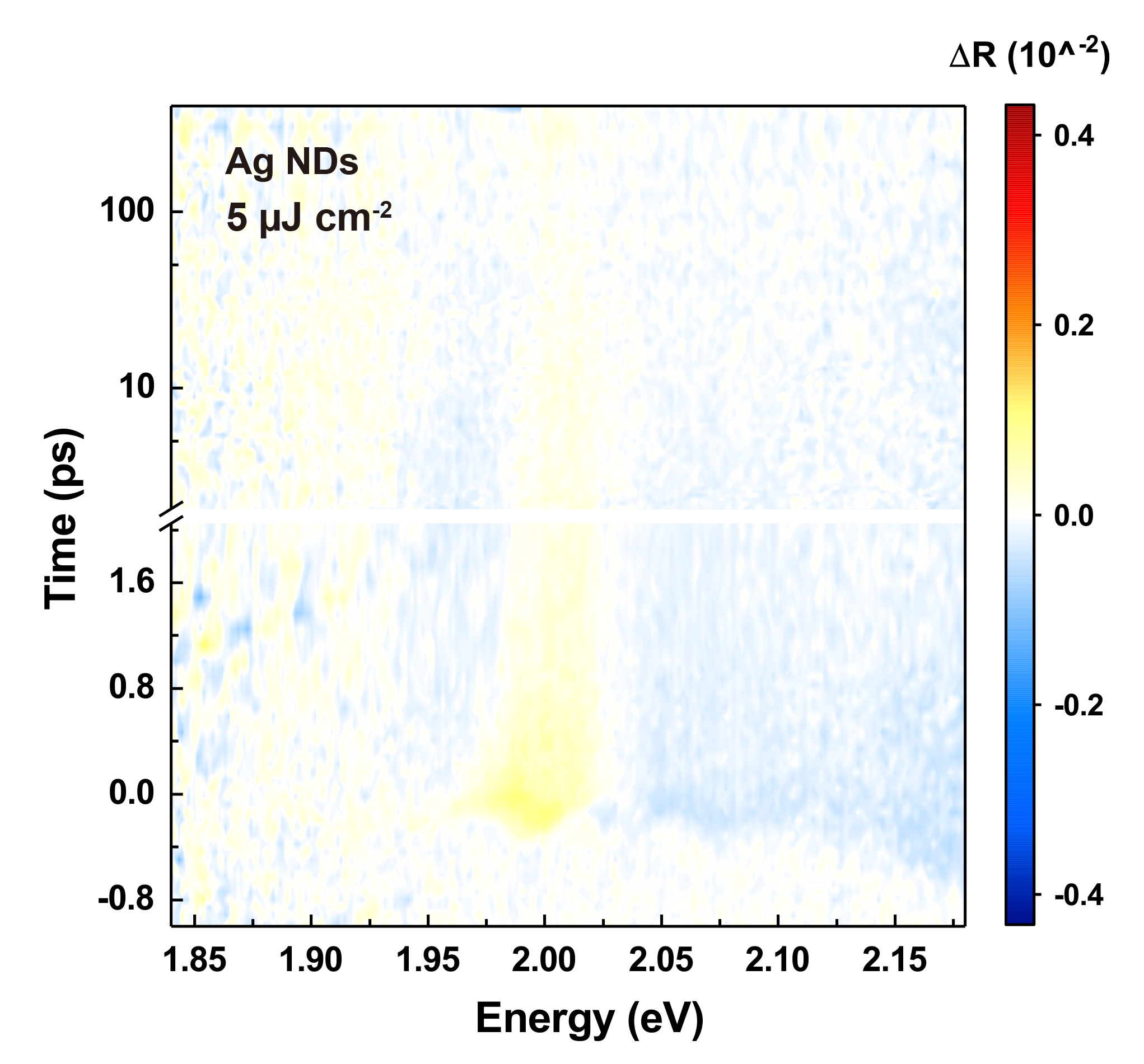


Figure S9. Time-resolved differential reflection spectra of individual Ag NDs under resonant pump excitation (2.0 eV) at incident fluences of 5 µJ cm^-2^.

**Supplementary Note 5: Time-resolved reflection spectra and E_UB_ ± E_LB_ dynamics of plexcitons under selective pump excitation circumstances.**

The whole time-resolved reflection spectra scan of plexcitons under 2.0 eV, 2.58eV, and 1.88 eV excitation are presented in Figure S10a-c, where the reflection spectra scan with 2.0 eV and 2.58 eV pump behaviors similarly, while no optical response is observed at excitation of 1.88 eV. The analogous phenomenon is also observed in the temporal evolution dynamics of E_UB_ ± E_LB_ displayed in Figure S10d-f. All these results indicate the nonlinearity of plexcitons is dominated by their excitonic part instead of the plasmonic part, which is consistent with the discussion in our main text.

Notably, the reflection spectra responses at 2.0 eV, 2.58eV excitation are not exactly same and there are still some minor differences between them (e.g., the intensity of UB plexciton). Here, we speculate these spectral differences are derived from the extra coulombic screening effect introduced by 2.58 eV excitation as 2.58 eV excitation pumps the high-energy free charge carriers rather than the excitons in the system^17^. Moreover, the E_UB_ ± E_LB_ dynamics at 2.0 eV excitation shows faster redshift compared with the dynamics at 2.58 eV. This discrepancy can be attributed to heat effect, which is largely absent in 2.58 eV excitation condition since 2.58 eV excitation cannot directly pump the plasmon.


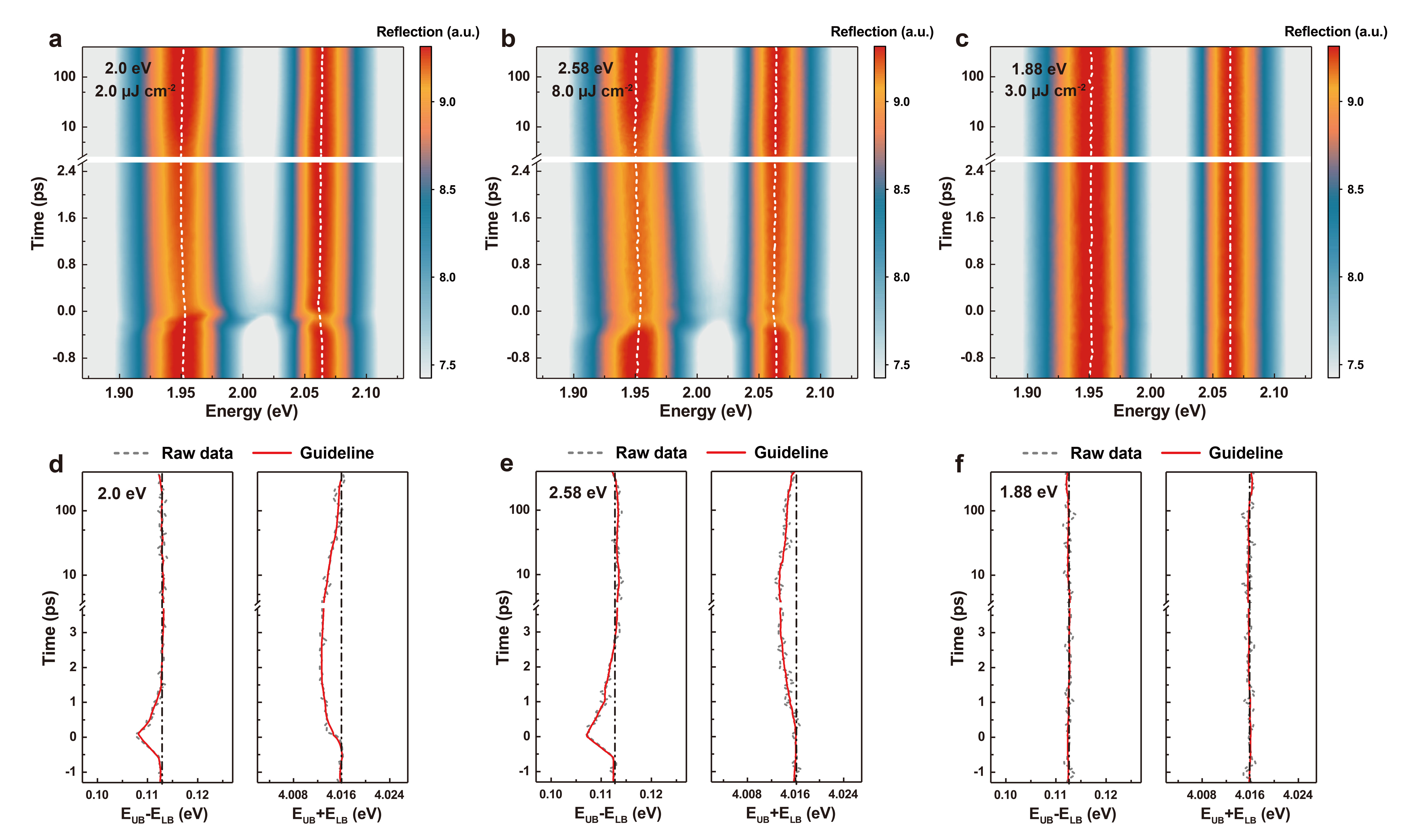


Figure S10. (a-c) Time-resolved reflection spectra scan of individual Ag ND-WS_2_ hybrid system under 2.0 eV, 2.58 eV, and 1.88 eV pump excitation at 2.0 µJ cm^-2^, 8.0 µJ cm^-2^, and 3.0 µJ cm^-2^. (d-f) Temporal evolution of E_UB_ ± E_LB_ acquired by subtracting and adding the E_UB_ and E_LB_ data in (a-c).

**Supplementary Note 6: COM fit of plexciton reflection spectra under excitation**

The COM fitting results of plexciton reflection spectra with and without EID effect at various incident fluences are shown in Figure S11. We can see that the measured data (red dashed lines) are well reproduced by COM fitting with the aid of EID effect (orange solid lines). Correspondingly, the extracted parameters are listed in Table S2. The apparent relative changes of exciton resonance linewidth ($\gamma_{x}$) and plasmon-exciton coupling strength ($g$) parameters indicate the presence of dephasing and saturation nonlinearity in plexcitons. Moreover, the nearly unchanged plasmon peak energy ($E_{p}$) and resonance linewidth ($\gamma_{p}$) parameters again certify the nonlinearity of plexcitons is from their excitonic component rather than plasmonic component (i.e., weak optical nonlinearity of plasmons).


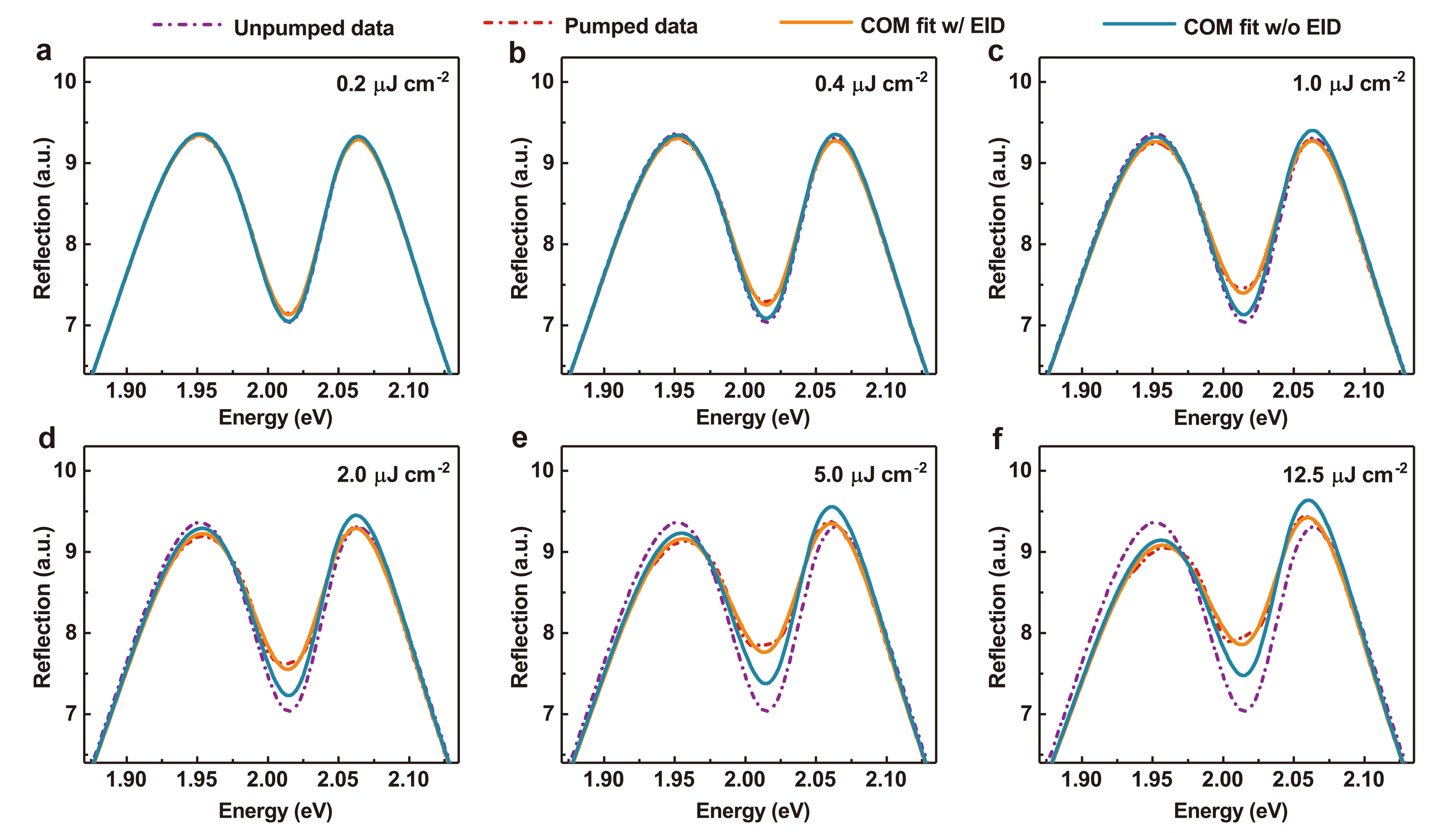


Figure S11. COM fitting results of the plexciton reflection spectra with and without the EID effect at pump excitation of (a) 0.2, (b) 0.4, (c) 1.0, (d) 2.0, (e )5.0, and (f) 12.5 µJ cm^-2^.

|  | $E_{x}$ (eV) | $\gamma_{x}$ (meV) | $E_{p}$ (eV) | $\gamma_{p}$ (meV) | $g$ (meV) | $\varphi$ (π) |
| --- | --- | --- | --- | --- | --- | --- |
| 0 µJ cm^-2^ | 2.0197 | 59.739 | 1.9966 | 269.85 | 92.564 | -0.0327 |
| 0.2 µJ cm^-2^ | 2.0195 | 61.869 | 1.9966 | 269.53 | 92.489 | -0.0333 |
| 0.4 µJ cm^-2^ | 2.0199 | 64.129 | 1.9964 | 269.82 | 91.870 | -0.0362 |
| 1.0 µJ cm^-2^ | 2.0198 | 67.021 | 1.9963 | 269.86 | 91.210 | -0.0393 |
| 2.0 µJ cm^-2^ | 2.0204 | 68.928 | 1.9962 | 270.88 | 89.499 | -0.0449 |
| 5.0 µJ cm^-2^ | 2.0209 | 71.353 | 1.9961 | 272.14 | 87.113 | -0.0546 |
| 12.5 µJ cm^-2^ | 2.0216 | 71.693 | 1.9961 | 273.88 | 86.465 | -0.0652 |

Table S2. COM fitting parameters obtained from Figure S11.

**Supplementary Note 7: Derivation of** $\boldsymbol{\Delta\gamma}_{\mathbf{x}}$**-**$\mathbf{g}$ **relationship from the bare WS_2_ excitons**

1. **Optical responses of bare WS_2_ excitons as a function of incident fluences**

The resonant excitation (i.e., 2.0 eV) pump-probe experiments of the bare ML WS_2_ system were performed here to experimentally establish the relationship of oscillator strength and resonance linewidth parameters in pure WS_2_ exciton, which is prepared for the theoretical derivation of ${\Delta\gamma}_{x}$-$g$ relationship in plexcitons. Figure S12 displays the measured pumped excitons reflection spectra at zero-time delay under various incident fluences. By fitting the measured excitons reflection spectra with Voigt functions, we can acquire the peak energy, oscillator strength (i.e., resonance area), and linewidth parameters correlation information of WS_2_ exciton at every incident fluence.


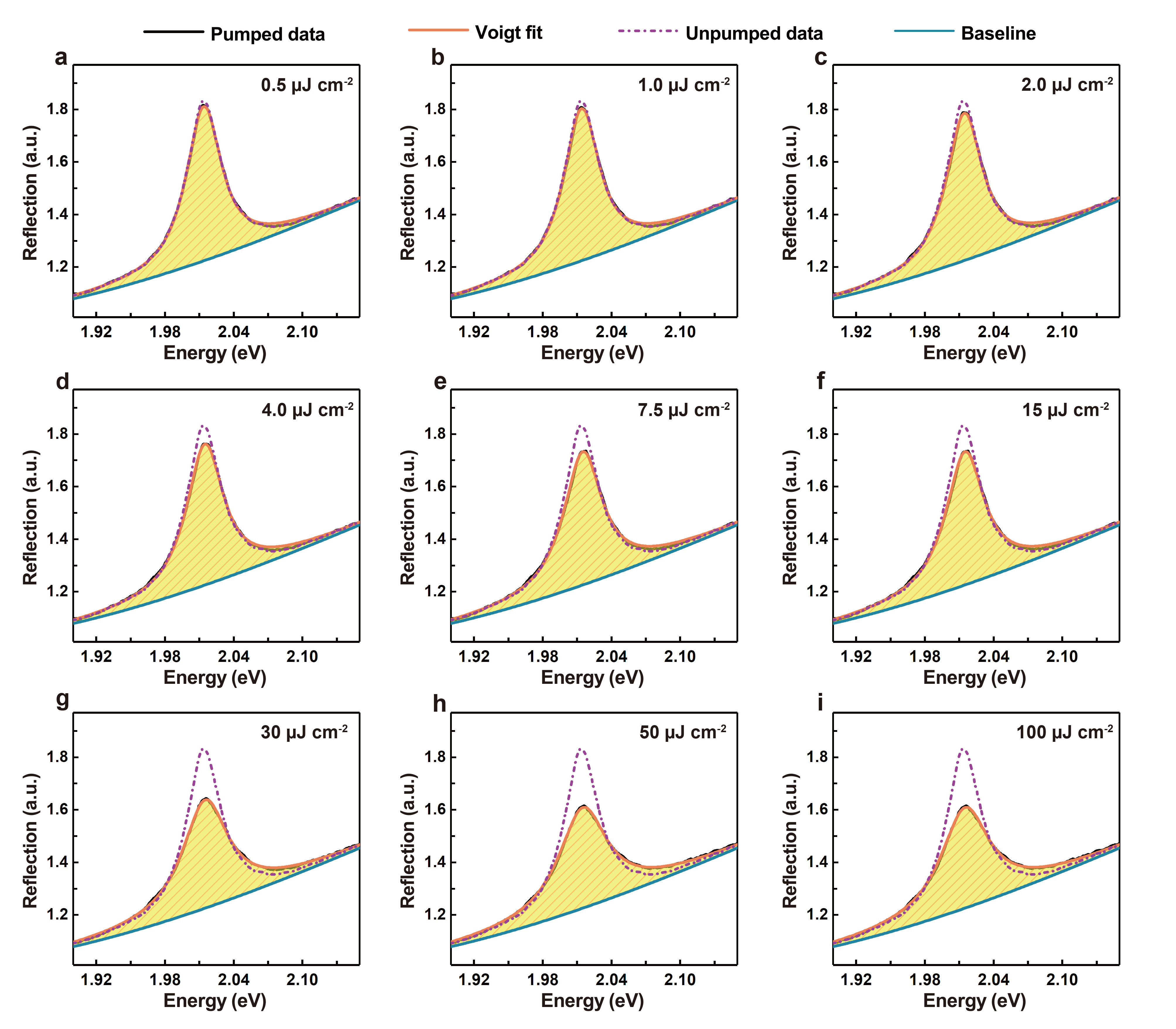


Figure S12. Bare WS_2_ exciton reflection spectra at zero time delay under 2.0 eV pump excitation with the fluences of (a) 0.5 µJ cm^-2^, (b) 1.0 µJ cm^-2^, (c) 2.0 µJ cm^-2^, (d) 4.0 µJ cm^-2^, (e )7.5 µJ cm^-2^, (f) 15 µJ cm^-2^, (g) 30 µJ cm^-2^, (h) 50 µJ cm^-2^, and (i) 100 µJ cm^-2^**.** The solid orange lines are the Voigt function fitting of the measured excitons reflection spectra with the assistance of blue solid baselines.

The extracted resonance peak energy ($E_{x}$), resonance area ($A)$, and resonance linewidth ($\gamma_{x}$) parameters as a function of incident fluences are plotted in Figure S13a-c. As can be seen, the blueshift of $E_{x}$ varies greatly at low incident fluences but approaches a saturation at high incident fluences, while the reduction of $A$ (i.e., saturation of oscillator strength) and broadening of $\gamma_{x}$ becomes more significant with increased incident fluences (> 15 µJ cm^-2^). This finding is consistent with the discussion in the main text, which states first-order exchange interaction between WS_2_ excitons (leading to the blueshift of resonance energy) works primally at large interparticle distance (low incident fluences) while the phase space filling effect and higher-order exciton-exciton interactions (e.g., excitation induced dephasing) become only important at short interparticle distance (high incident fluences). Furthermore, Figure S13d records the kinetics of WS_2_ exciton (probed at $E_{x}$) at these pump incident fluences. The faster relaxation dynamics at higher incident fluences implies exciton-exciton collisions become quite efficient at large excitons density accompanied by the emergence of higher-order many-body effect^18,19^.


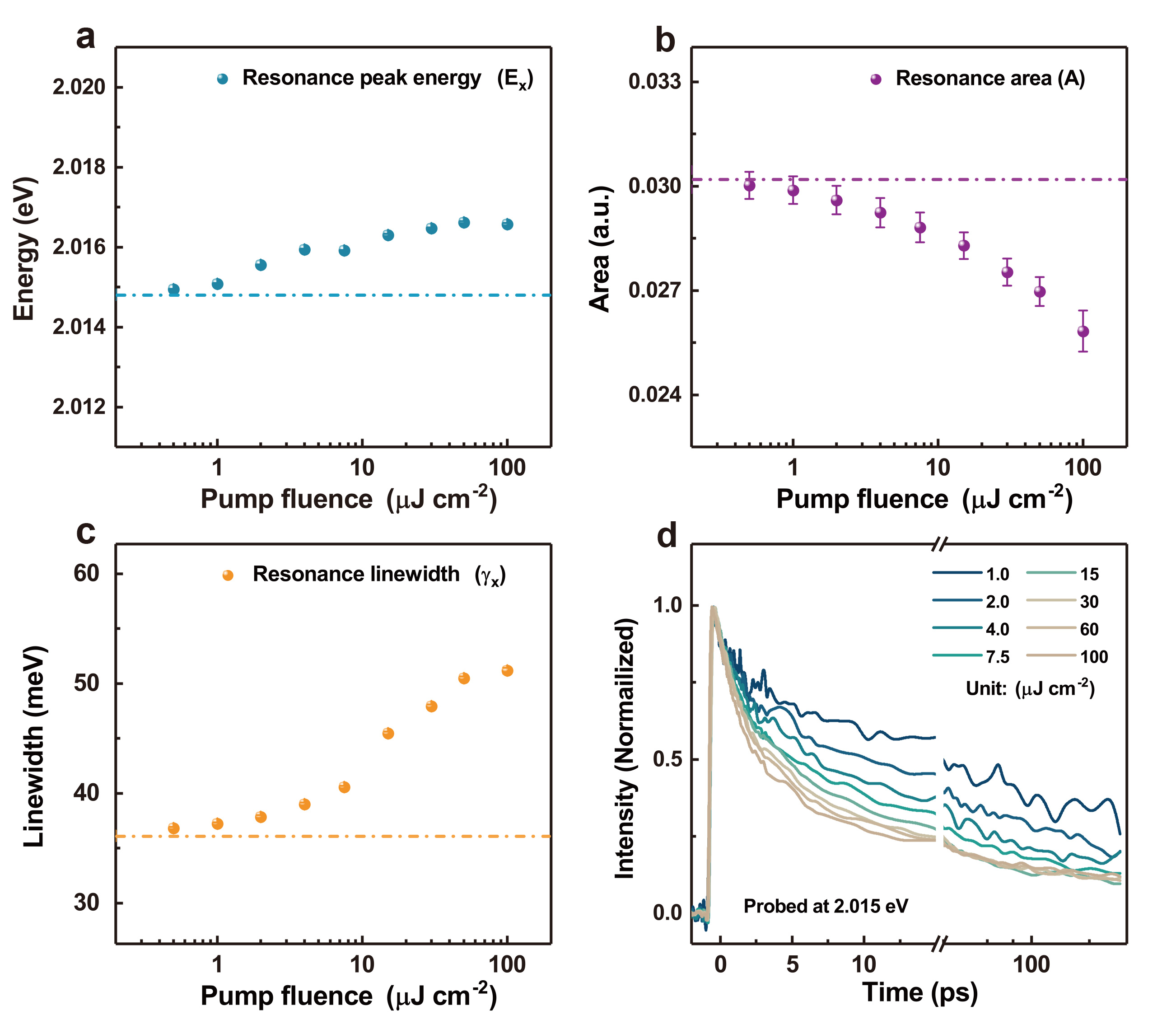


Figure S13. Pump fluences dependent parameters evolution of (a) exciton resonance peak energy ($E_{x}$), (b) exciton resonance area ($A$), and (c) exciton resonance linewidth ($\gamma_{x}$). The dash-dot lines denote the initial values of parameters at zero pump incident fluences. (d) Bare WS_2_ exciton kinetics probes at 2.015 eV under different excitation fluences.

1. **Derivation of** $\boldsymbol{\Delta\gamma}_{\mathbf{x}}$**-**$\mathbf{g}$ **relationship**

Since the oscillator strength (reflected by the spectral integral of exciton resonance. i.e., resonance area $A$) and resonance linewidth ($\gamma_{x}$) parameters relationship of bare WS_2_ excitons has been experimentally acknowledged at every incident fluence (as shown in Figure S13b, c), we can theoretically derive the ${\Delta\gamma}_{x}$-$g$ relationship in plexcitons according to the mathematical expression of $g \propto\sqrt{A}$ ^20^. The estimated $g$ values in plexcitons are calculated by the relative change of resonance area ($g_{0}\cdot\sqrt{A/{A_{0}}}$), where $g_{0}$ and $A_{0}$ represent the initial plexciton coupling strength and exciton oscillator strength under zero pump incident fluence, respectively. As a result, the processed ${\Delta\gamma}_{x}$-$\sqrt{A/{A_{0}}}$ relationship (i.e., ${\Delta\gamma}_{x}$-$g$ relationship) are displayed in Figure S14 (black dots). Phenomenologically, we fit the ${\Delta\gamma}_{x}$-$\sqrt{A/{A_{0}}}$ relationship with a well-established model for classical 2D excitons^21-23^:

$$f(n)=f_{0}/(1+n/n_{s}) \left( S10 \right)$$

$$\gamma_{x}\left( n \right)=\gamma_{0}+\alpha(n)\cdot n \left( S11 \right)$$

$$\alpha\left( n \right)=\alpha_{0}/(1+n/n_{x}) \left( S12 \right)$$

Where Equation S10 is the conventional exciton oscillator strength saturation model, Equation S11 and S12 represent optimized linewidth broadening model (due to EID) with a density-dependent coefficient $\alpha\left( n \right)$. $f_{0}$ and $\gamma_{0}$ are the initial exciton oscillator strength and resonance linewidth without pump excitation. $n$ is the density of the photoexcited exciton, $n_{s}$ and $n_{x}$ are the saturation exciton densities for oscillator strength and linewidth broadening coefficient. The obtained fitting curve of the ${\Delta\gamma}_{x}$-$\sqrt{A/{A_{0}}}$ relationship is plotted in Figure S14 (red solid line), which is used in our main text to prove the accuracy of the COM fitting results of pumped plexcitons reflection spectra.


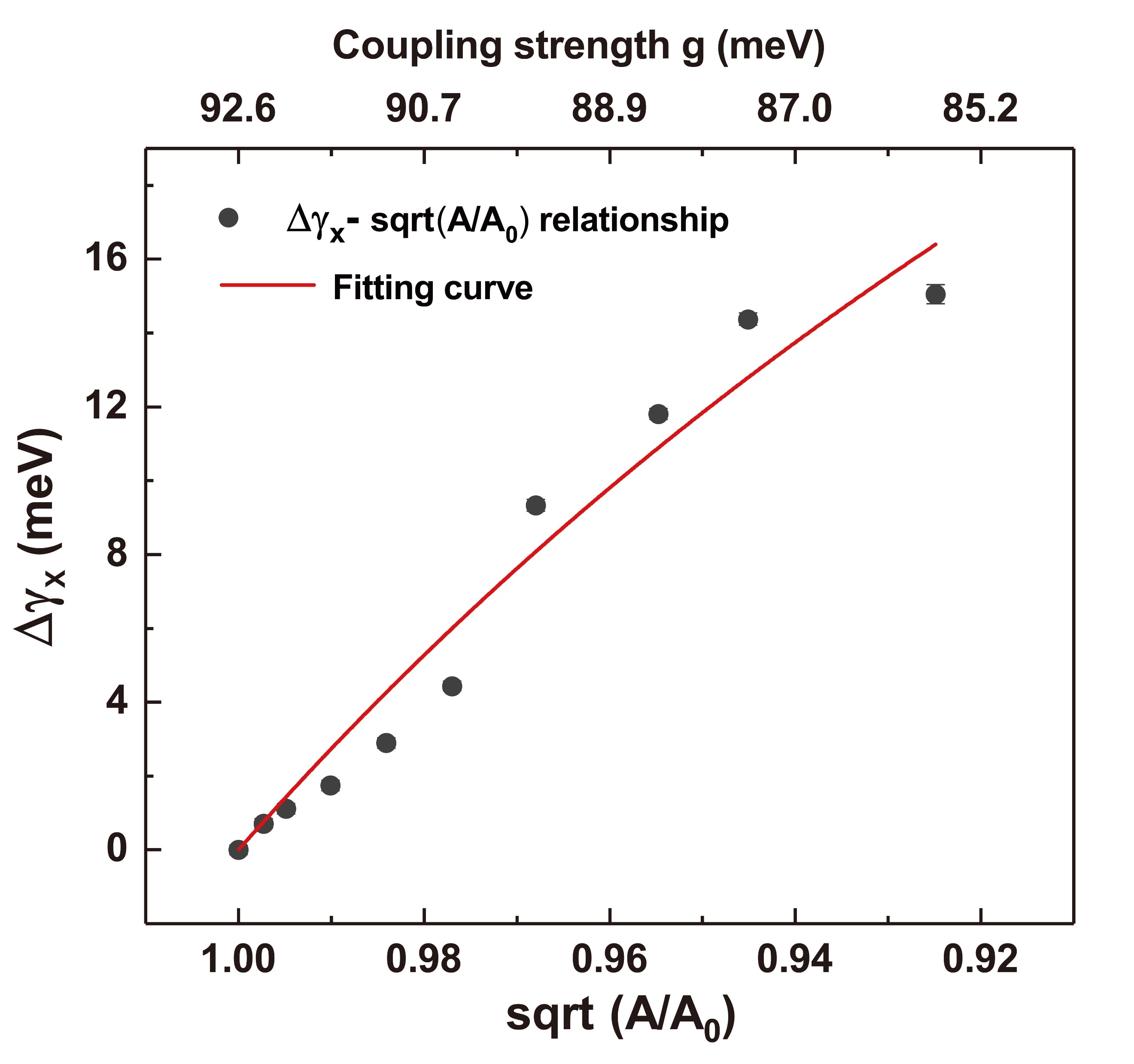


Figure S14. ${\Delta\gamma}_{x}$-$g$ relationship derived from the bare WS_2_ excitons. The red solid line illustrates the fitting result of ${\Delta\gamma}_{x}$-$g$ relationship via using a classical 2D excitons model.

**Supplementary Note 8: The evaluation of plexcitons nonlinearity**

1. **Estimation of plexcitons nonlinearity from other microcavity polaritons nonlinearities**

As we mentioned in the main text, the specific coefficient of plexcitons nonlinearity is hard to be reckoned in our complex system due to the difficulty to accurately gauge the excited plexcitons density. However, a comparison of plexcitons nonlinearity with other polaritons nonlinearity will be helpful for us to have a rough estimation of the interaction constant of plexcitons nonlinearity in our work. Thus, Table S3 summarizes a list of TMD-based polaritons nonlinearity reported in recent years.

In this table, the polariton nonlinearities are mainly classified in terms of the exact type of polaritons (i.e., exciton-polaritons, trion-polaritons, polaron-polaritons, Moiré exciton-polaritons, Rydberg exciton-polaritons, interlayer exciton-polaritons, and plasmon-exciton-polaritons) and the key mechanism (i.e., exchange, saturation, and dephasing interaction) governing the nonlinear responses. After a comprehensive comparison, we speculate here that the coefficient of plexcitons nonlinearity in our work is most likely to be close to the coefficient of neutral exciton-polaritons nonlinearity extracted at high power excitation, and the corresponding constants of plexcitons nonlinearity (exchange and saturation) are most probably at the range of 10^-3^~10^-1^ µeV∙µm^2^ (refer to the literature [24, 26]) as the nonlinearity of plexcitons is originated from their neutral exciton component and the small volume (optical confinement from LSPR) of plexcitonic mode leads to a fairly high plexcitons density generated surrounding the Ag ND, just similar to the case where dielectric microcavity exciton-polaritons are excited under intense laser fluence.

It should be noted that although the coefficient of plexcitons nonlinearity is on the same level as the neutral exciton-polaritons and not as prominent as the other kinds of polaritons (trion, polaron, Moiré exciton, Rydberg exciton, interlayer exciton), the nonlinear performances of Ag ND-WS_2_ plexcitonic system are still considerable since the optical confinement (beyond diffraction limit) of plexcitonic mode results in naturally high excited plexcitons density in the system, which facilitates the occurrence of the nonlinear responses (considering that the ultimate nonlinear response magnitude of Ag ND-WS_2_ device is dependent on the product of nonlinearity coefficient and the plexcitons density).

| Year | Materials types | Resonators types | Polaritons types  (Rabi splitting) | Nonlinearity types | Coefficient  (µeV∙µm^2^) | Testing temperature |
| --- | --- | --- | --- | --- | --- | --- |
| 2018^24^ | ML  WS_2_ | Bragg mirror | Exciton-polaritons  (43.4 meV) | Exchange ($\beta_{x}$) | 6.0 × 10^-2^ | 295 K |
|  |  |  |  | Saturation ($\beta_{s}$) | 5.0 × 10^-3^ |  |
| 2020^25^ | ML  MoSe_2_ | Photonic crystal slab | Exciton-polaritons  (27.4 meV) | Exchange ($\beta_{x}$) | 1.0 ± 0.4 | 7 K |
| 2020^26^ | ML  MoSe_2_ | Distributed Bragg reflector | Trion-polaritons  (5.8 meV) | Saturation ($\beta_{x}$) | 37 ± 3.0 | 4 K |
|  |  |  | Exciton-polaritons  (17.2 meV) | Exchange ($\beta_{x}$)/ Saturation ($\beta_{s}$) | 1.0×10^-2^ ~ 2.0 |  |
| 2020^27^ | ML  MoSe_2_ | Distributed Bragg reflector | Polaron-polaritons  (2.6 meV) | Saturation ($\beta_{x}$) | 0.5 | 4.2 K |
|  |  |  | Exciton-polaritons  (14.0 meV) | Exchange ($\beta_{x}$) | 1.0 × 10^-2^ |  |
| 2021^28^ | ML  MoSe_2_ | Distributed Bragg reflector | Exciton-polaritons  (28.0 meV) | Exchange ($\beta_{x}$) | 2.2 ± 1.6 | 127 K |
|  |  |  |  | Saturation ($\beta_{s}$) | 2.16 ± 0.5 |  |
| 2021^29^ | MLWS_2_/  MoSe_2_ | Planar microcavity | Moiré exciton-polaritons  (10.1 meV) | Saturation ($\beta_{s}$) | 6.0×10^-2^ ~ 5.0 | 70 K |
|  |  |  | Exciton-polaritons  (17.1 meV) | Exchange ($\beta_{x}$)/ Saturation ($\beta_{s}$) | 2.0×10^-2^ |  |
|  |  |  |  | Dephasing ($\beta_{d}$) | N/A |  |
| 2021^30^ | ML  WSe_2_ | Distributed Bragg reflector | Rydberg exciton-polaritons  (7.5 meV) | Saturation ($\beta_{s}$) | 46.4 ± 13.9 | 15 K |
|  |  |  | Exciton-polaritons  (27.0 meV) | Exchange ($\beta_{x}$)/ Saturation ($\beta_{s}$) | 10.0 ± 4.2 |  |
| 2021^31^ | Bilayer  MoS_2_ | Distributed Bragg reflector | Interlayer  exciton-polaritons  (21.4 meV) | Exchange ($\beta_{x}$)/ Saturation ($\beta_{s}$) | 2.0 ~100.0 | 7 K |
|  |  |  | Exciton-polaritons  (40.4 meV) | Exchange ($\beta_{x}$)/ Saturation ($\beta_{s}$) | 6.6 ~ 10.0 |  |
| This Work | ML  WS_2_ | Ag ND | Plasmon-exciton-polaritons  (112.3 meV) | Exchange ($\beta_{x}$) | 10^-3^ ~ 10^-1^ | 295 K |
|  |  |  |  | Saturation ($\beta_{s}$) | 10^-3^ ~ 10^-1^ |  |
|  |  |  |  | Dephasing ($\beta_{d}$) | N/A |  |

Table S3. List of TMD-based microcavity polaritons nonlinearity reported from other works and a rough estimation of plexcitons nonlinearity in our work.

1. **Comparison of nonlinearity of hybrid Ag-WS_2_ plexcitons and bare WS_2_ excitons as a function of pump fluence**

Since extracting the coefficient of the plexcitons nonlinearity is a rather challenging task in our work and the value of nonlinearity coefficient cannot be used to intuitively reflect the ultimate nonlinear response performance of the Ag-WS_2_ hybrid device, a comparison of the nonlinearity of hybrid Ag-WS_2_ plexcitons with the nonlinearity of bare WS_2_ excitons as a function of pump fluence would be helpful for us to better quantitively evaluate the magnitude of plexcitons nonlinearity and highlight its significance. Thus, we systematically compare the nonlinear response signals of the Ag-WS_2_ plexciton system and WS_2_ excitons system in terms of two kinds of nonlinearity (i.e., the saturation nonlinearity, $\beta_{s}$, and the dephasing nonlinearity, $\beta_{d}$) in Figure S15. Parameters like normalized coupling/oscillator strength (defined as $g/{g_{0}}$ for Ag-WS_2_ plexcitons and $\sqrt{A/{A_{0}}}$ for bare WS_2_ excitons with relationship $g/{g_{0}}\propto\sqrt{A/{A_{0}}}$) and linewidth broadening (${\Delta\gamma}_{x}$) are utilized to characterize the magnitude of saturation and dephasing nonlinearities, respectively. It can be observed that, for the same magnitude of produced nonlinear responses (i.e., coupling/oscillator strength bleaching in Figure S15a and linewidth broadening in Figure S15b), the pump pulse fluences consumed by the hybrid Ag-WS_2_ plexcitons system are almost average 10 times smaller than the ones used in the bare WS_2_ excitons system. Although the times are changing at every pump fluences potentially due to the varying nonlinear interaction constants at different plexcitons density, an average tenfold difference is most reasonable when accounting for both the cases of saturation and dephasing nonlinearities.


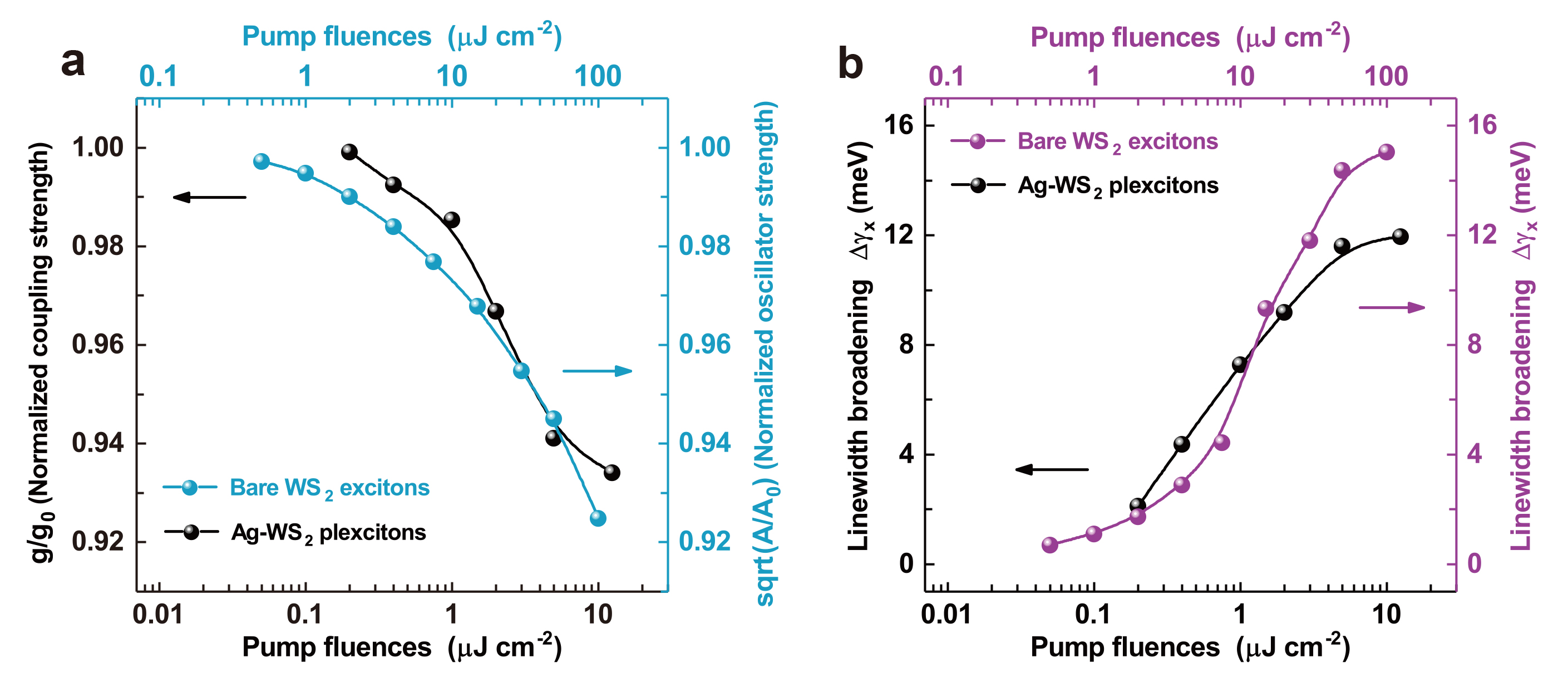


Figure S15. Comparison of nonlinear responses of Ag-WS_2_ plexcitons and WS_2_ excitons as a function of pump fluences in terms of (a) saturation and (b) dephasing nonlinearities. The fluences axis of excitons is magnified by a factor of 10 compared to that of plexcitons, for better comparison.

**Supplementary Note 9: Plexcitons nonlinearity at increased coupling strengths**

Since plexcitons nonlinearity is only studied under moderate plasmon-exciton coupling strength condition in the main text, it is very necessary to explore the applicability of our conclusion along with the increase of the coupling strength. In order to obtain the Ag ND-WS_2_ plexcitonic devices with increased coupling strengths, three WS_2_ samples of different layer numbers, i.e., monolayer (1L), bilayer (2L), and trilayer (3L), were firstly prepared by mechanically stacking the CVD-grown single-layer WS_2_ onto a same glass substrate three times via wet transfer procedures. Subsequent annealing process (300 °C for 3 hours in a vacuum environment) ensures the good quality of our sample. Figure S16 demonstrates the measured excitons reflection spectra of the fabricated 1L, 2L, and 3L WS_2_. We can see that the exciton reflection signal is enhanced with the increase of WS_2_ layer thickness. Simultaneously, the redshift of exciton energy and broadening of resonance linewidth are also observed in multi-layer WS_2_ relative to single-layer WS_2_ due to the interlayer coupling effect.


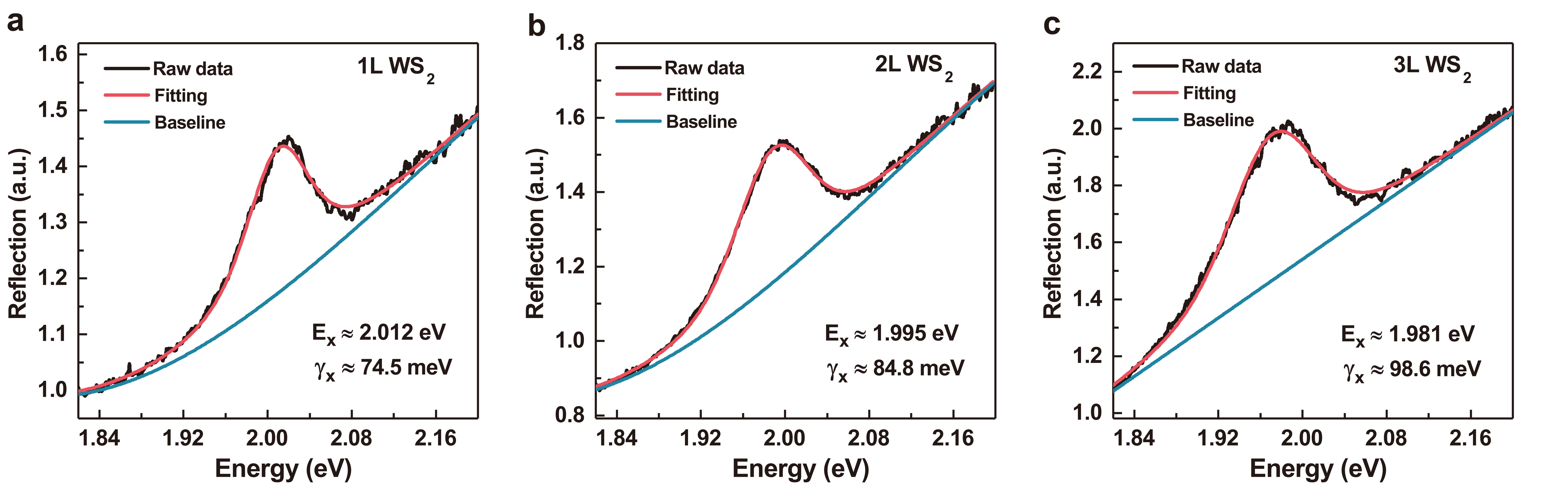


Figure S16. (a-c) Exciton reflection spectra of WS_2_ at different thicknesses. The measured data (black lines) are well fitted by the Voigt curves (red lines) with the help of blue baselines.

By integrating the WS_2_ of different layer numbers ($N$) with the Ag NDs, we obtain the Ag ND-WS_2_ plexcitonic devices with different coupling strength ($g$), since $g$ is theoretically proportional to $\sqrt{N}$^32^. Figure S17a-c shows the measured reflection spectra results of Ag ND-WS_2_ at increased layer numbers. As can be seen, the coupling strength is enhanced by varying the number of layers from 1L to 3L. However, this enhancement is less than the theoretically expected value because in-plane dipole moments of WS_2_ decrease in thicker multilayers. Besides, the coupling strength (85.1 meV) of Ag ND-1L WS_2_ here is smaller than the one (92.6 meV) reported in the main text, which is likely caused by the sample damage from multiple wet transfer procedures. Finally, we conducted resonant pump-probe measurements on these Ag ND-WS_2_ plexcitons systems in Figure S17d-f for acquiring the ultrafast optical response information of plexcitons at increased coupling strength.


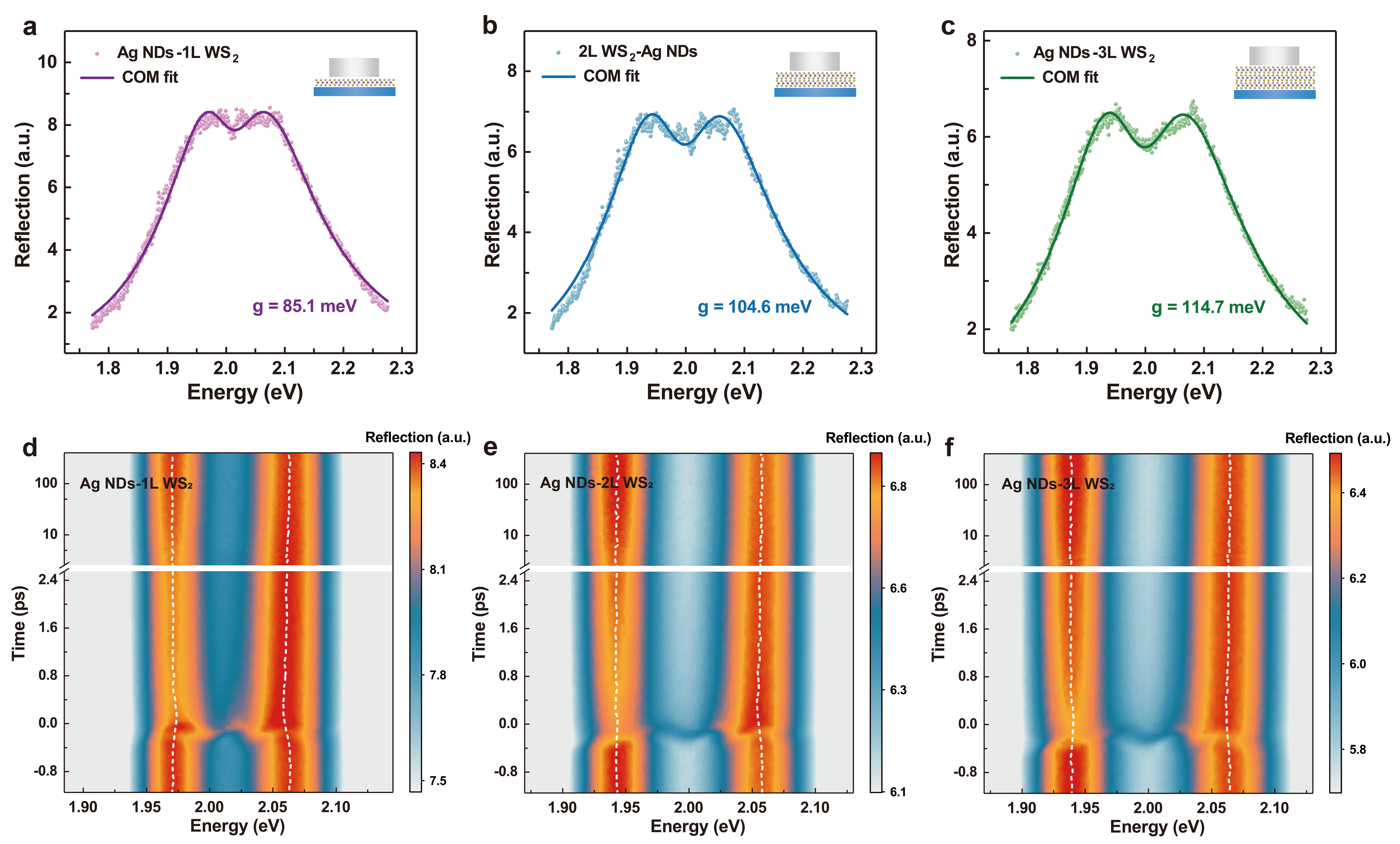


Figure S17. Thickness-dependent (a-c) steady-state reflection spectra and (d-f) time-resolved reflection spectra scan of Ag ND-WS_2_ plexcitons under pump incident fluences of 2.0 µJ cm^-2^.

The plexcitons reflection spectra of Figure S17d-f at t = 100 fs are correspondingly extracted in Figure S18a-c to investigate the plexcitons nonlinearity at increased coupling strength. It can be clearly seen that dephasing nonlinearity (i.e., broadening of resonance linewidth) and saturation nonlinearity (i.e., reduction of spectral splitting) dominate the plexcitons nonlinearity in all cases. This fact verifies the [universality](http://www.baidu.com/link?url=6Ti6yGKuVd-CEuziH4BJTfqK2bE_CBaOAuzxpVUV4WwtSj5mlzNuInJcMA0Q9bcVQdtvliI5hEdoL16Or2VL1ssg2989KaFg3_m4pt_PFUW) of our conclusion that plexcitons nonlinearity is governed by excitation-induced dephasing and phase-space filling mechanisms. The much more obvious dephasing nonlinearity in the 2L and 3L Ag ND-WS_2_ systems compared with the 1L Ag ND-WS_2_ system suggests interlayer coupling effect (e.g., interlayer excitons assisted EID effect^33^) also play an important role in determining the whole plexcitons nonlinearity.


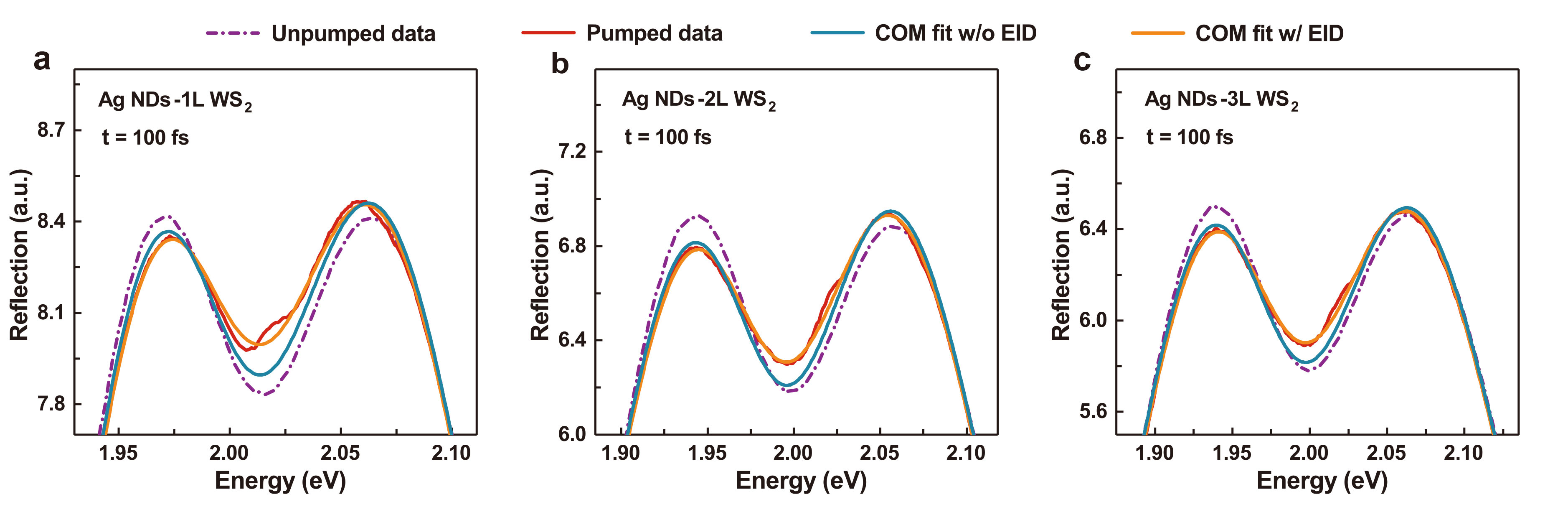


Figure S18. (a-c) COM fitting results of the plexciton reflection spectrum at increased coupling strength with (orange solid line) and without (blue solid line) the EID effect.

### **Supplementary Note 10: Tunable plexcitons for designed ultrafast optical nonlinearity**

- 1. **Ultrafast optical responses of Ag ND-WS_2_ samples with different Al_2_O_3_ thickness**

### The complete time-resolved reflection spectral scans and selected spectral signals at t = 100 fs of Ag ND-WS_2_ sample with different Al_2_O_3_ thickness are displayed in Figure S19.


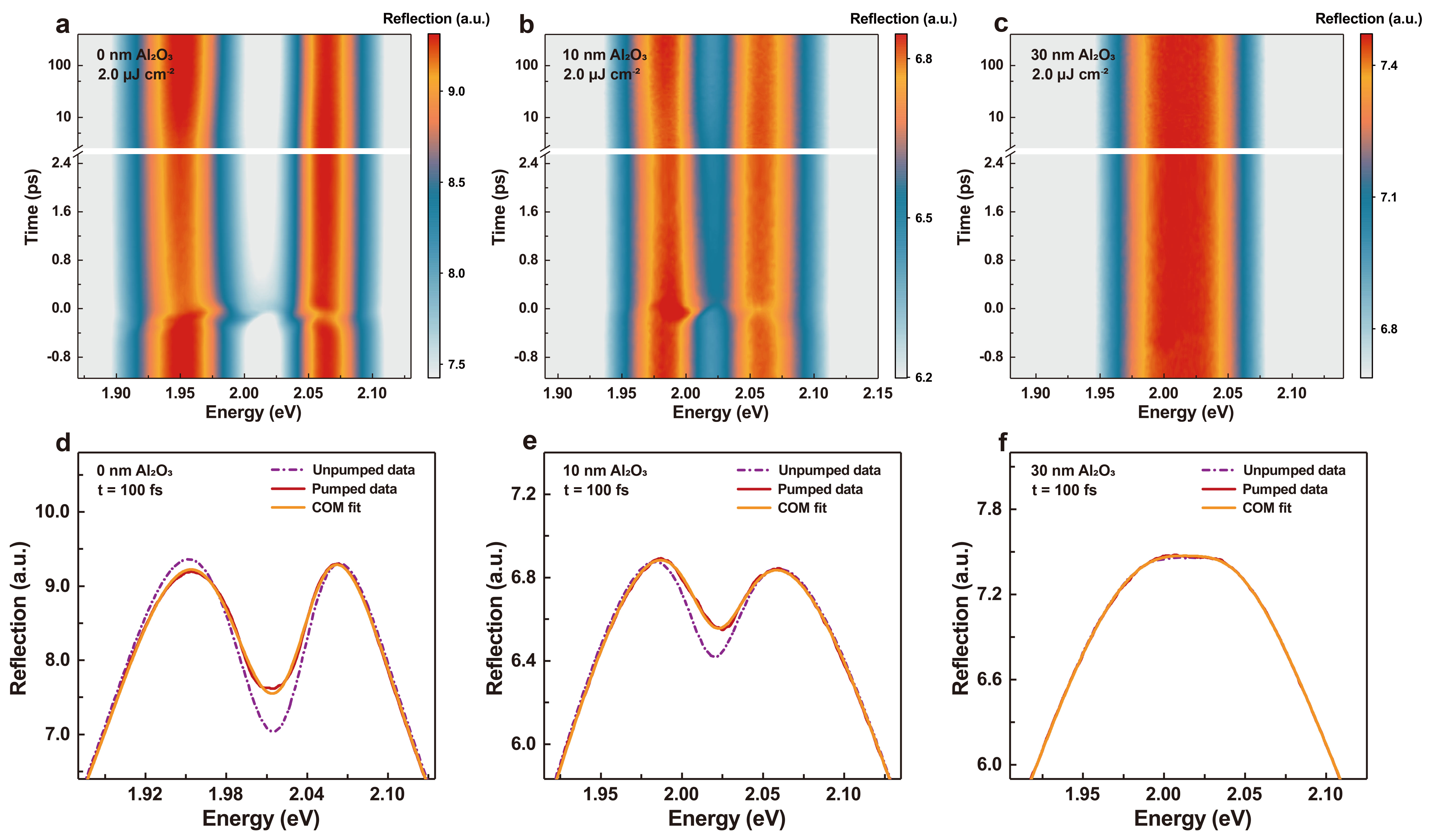


Figure S19. Time-resolved reflection spectra scan of Ag ND-WS_2_ samples with (a) 0 nm, (b) 10 nm, and (c) 30 nm Al_2_O_3_ interlayer under 2.0 µJ cm^-2^ resonant excitation. Corresponding reflection spectra at = 100 fs are shown in (d), (e), and (f), respectively.

- 1. **Ultrafast optical responses of Ag ND-WS_2_ samples with distinct disk diameters**

The complete time-resolved reflection spectral scans and selected spectral signals at t = 100 fs of Ag ND-WS_2_ sample with distinct disk diameters are shown in Figure S20.


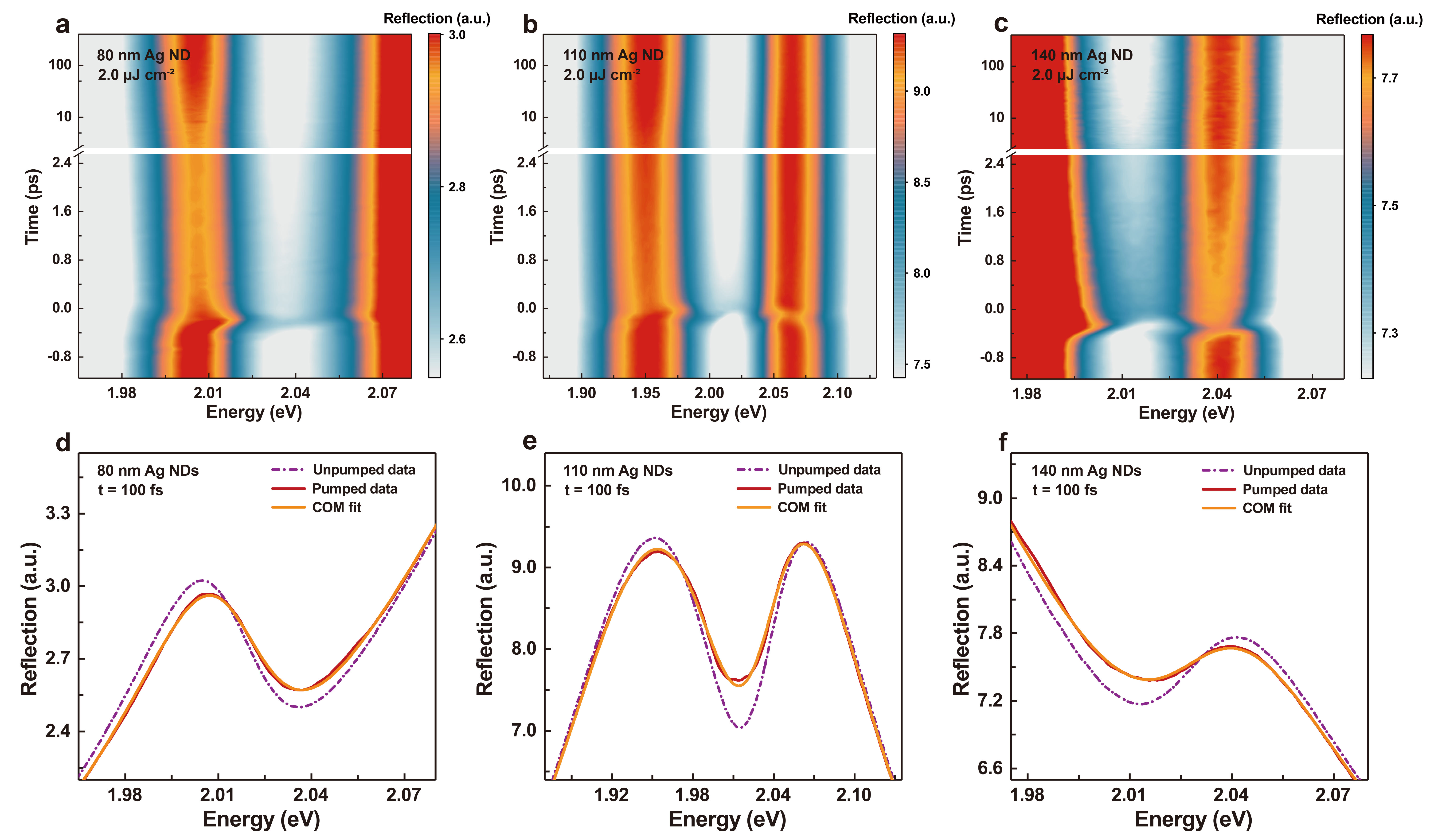


Figure S20. Time-resolved reflection spectra scan of Ag ND-WS_2_ samples with (a) 80 nm, (b) 110 nm, and (c) 140 nm disk diameters under 2.0 µJ cm^-2^ resonant excitation. Corresponding reflection spectra at = 100 fs are shown in (d), (e), and (f), respectively

### The successive changes of differential reflection spectra as a function of disk diameters are displayed in Figure S21. We can see the PA signal continues shifting to the low energy direction as the plasmon resonance energy decreases. At the same time, the PB signal also changes from the red side of PA signal to the blue side of PA signal. These phenomena are caused by the changes of constructive enhancement and destructive suppression positions at different plasmon-exciton tuning conditions.


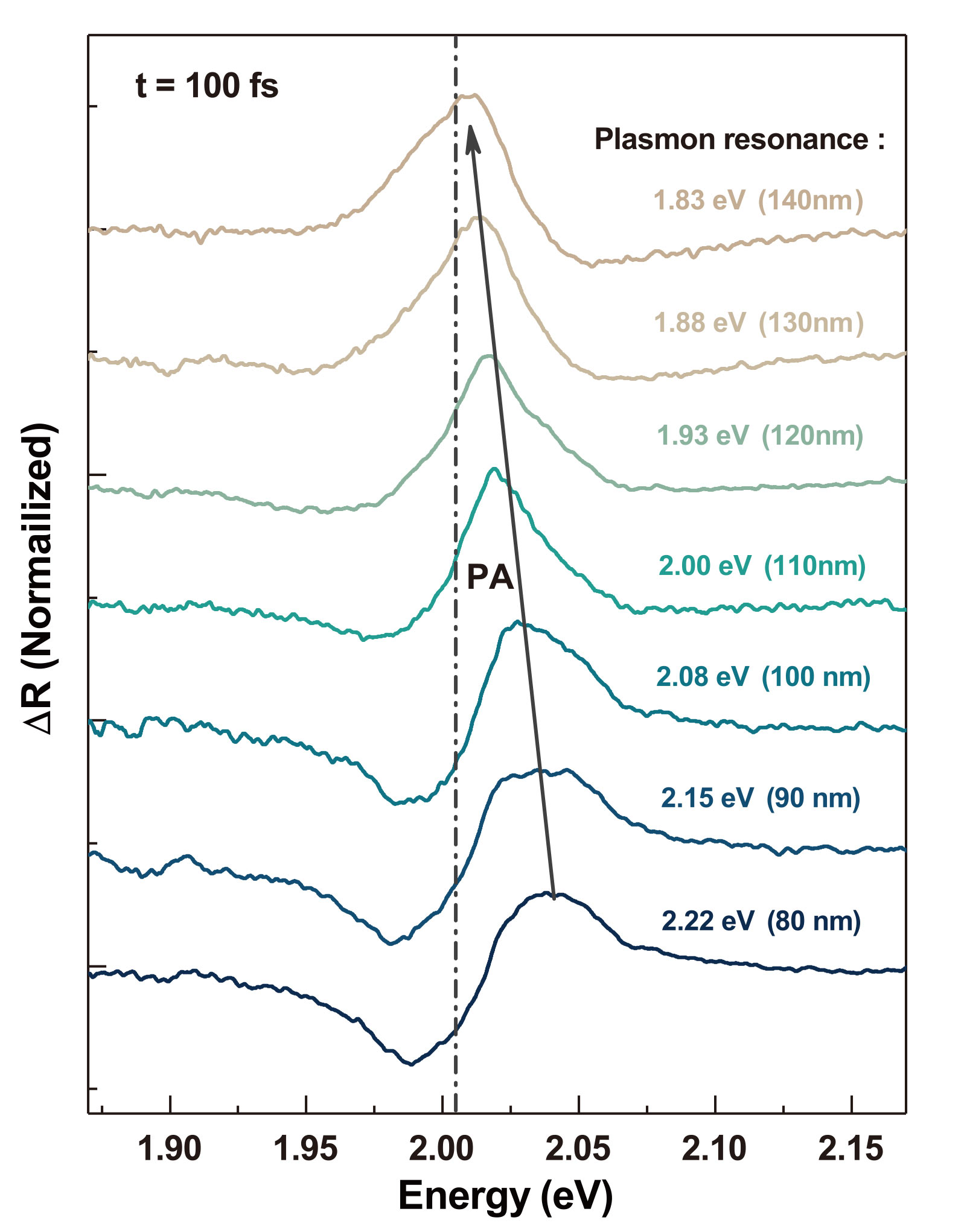


Figure S21. Differential reflection spectra of Ag ND-WS_2_ samples at t =100 fs with continuously-varying disk diameters under 2.0 µJ cm^-2^ resonant excitation.

- 1. **I-scan measurements of individual monolayer WS_2_, Ag nanodisks, and substrate**


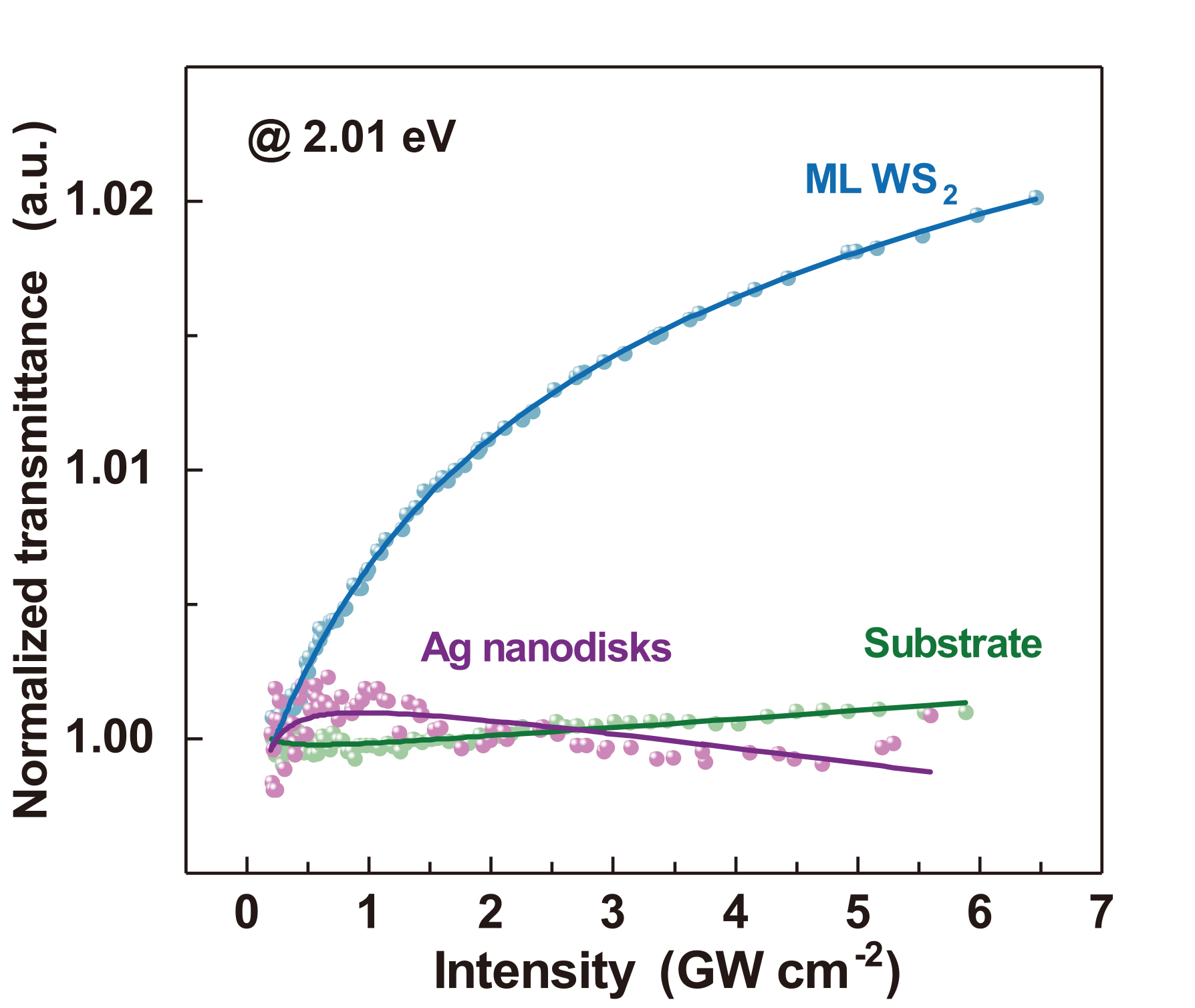


Figure S22. The nonlinear absorption properties of bare ML WS_2_ substrate, Ag NDs, and substrate. As can be seen, the WS_2_ demonstrates a typical saturable absorption response while Ag NDs and substrate exhibit barely any nonlinear response, which proves the accuracy of our I-scan results.

### **Supplementary Note 11: The influence of plasmonic hot electrons transfer**

### If there is any plasmonic hot electrons transfer in our plexcitonic system, we expect the time-resolved differential reflection spectra of Ag ND-WS_2_ sample will show (i) changes in spectral shapes and temporal dynamics due to the refilling of excited-state populations in ML WS_2_^34^, and (ii) ultrashort broad bleaching signals (i.e., bandgap renormalization with population inversion) at lower photon energies (< 1.85 eV) due to the coherent doping of plasmonic hot electrons^35^. Figure S23 shows the ultrafast differential responses of Ag ND-WS_2_ sample with and without dielectric interlayers, in which 2 nm Al_2_O_3_ was adapted to reduce the transfer probability of plasmonic electrons in the metal-semiconductor interface. No obvious spectral feature changes that represent the existence of plasmonic hot electrons transfer are observed in Figure S23a, b, which implies plasmonic hot electrons transfer plays a minor role in our results (within the range of pump incident fluences used in our measurements).

### Moreover, we have already performed below bandgap excitation (1.88 eV) measurements on Ag ND-WS_2_ sample in Supplementary Note 5, where no optical response signals were detected at this case. This result further indicates plasmonic hot electrons transfer is not enough efficient^36^ in our sample to affect the measured plexcitons nonlinearity.

###
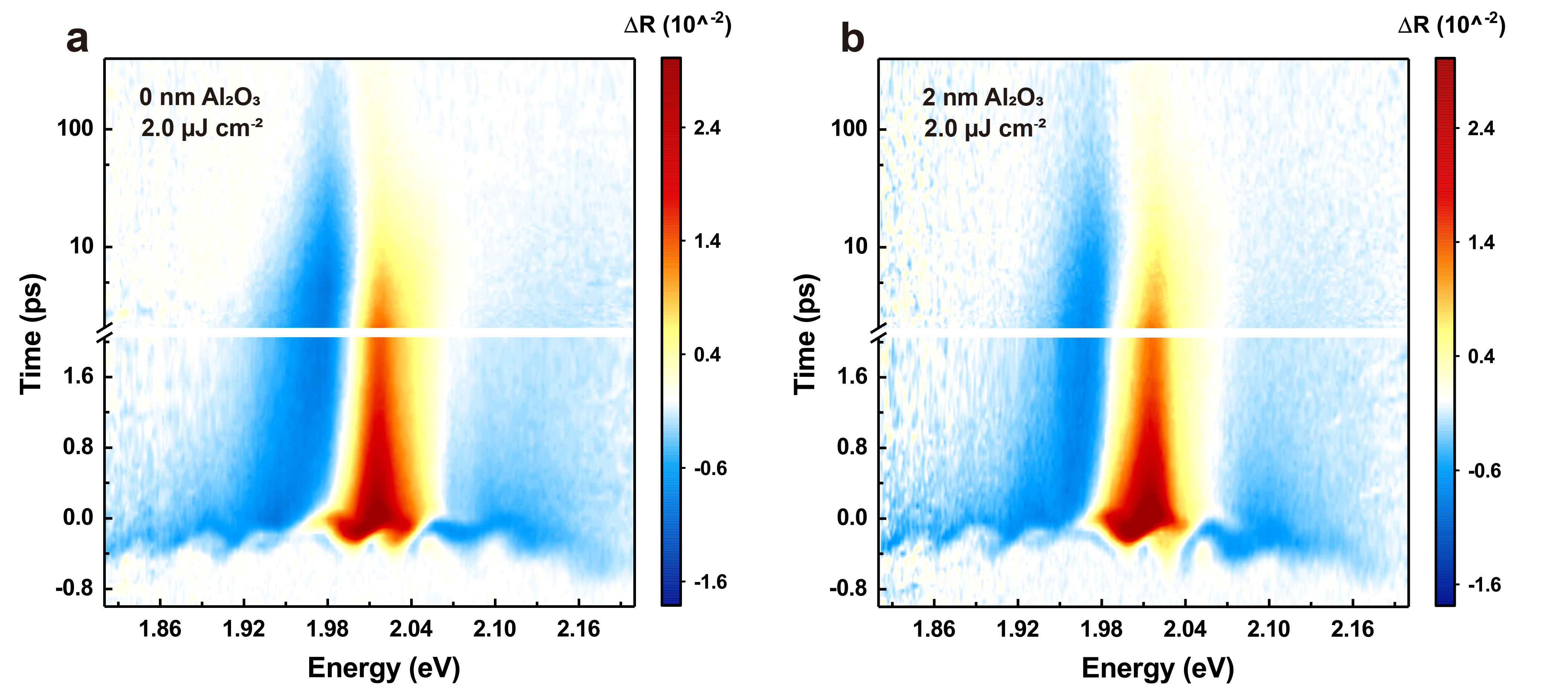


### Figure S23. Time-resolved differential reflection spectra of the Ag ND-WS_2_ plexcitons under 2.0 µJ cm^-2^ resonant excitation (a) without Al_2_O_3_ interlayer and (b) with 2 nm Al_2_O_3_ interlayer.

**References**

1 Rechberger, W. *et al.* Optical properties of two interacting gold nanoparticles. *Optics Communications* **220**, 137-141, (2003).

2 Kravets, V. G., Kabashin, A. V., Barnes, W. L. & Grigorenko, A. N. Plasmonic Surface Lattice Resonances: A Review of Properties and Applications. *Chemical Reviews* **118**, 5912-5951, (2018).

3 Cherqui, C., Bourgeois, M. R., Wang, D. & Schatz, G. C. Plasmonic Surface Lattice Resonances: Theory and Computation. *Accounts of Chemical Research* **52**, 2548-2558, (2019).

4 Novotny, L., J American Journal of Physics. Strong coupling, energy splitting, and level crossings: A classical perspective. **78**, 1199-1202, (2010).

5 Satpathy, S., Roy, A. & Mohapatra, A. Fano interference in classical oscillators. *European Journal of Physics* **33**, 863-871, (2012).

6 Wu, X., Gray, S. K. & Pelton, M. Quantum-dot-induced transparency in a nanoscale plasmonic resonator. *Opt. Express* **18**, 23633-23645, (2010).

7 Shah, R. A., Scherer, N. F., Pelton, M. & Gray, S. K. Ultrafast reversal of a Fano resonance in a plasmon-exciton system. *Physical Review B* **88**, 075411, (2013).

8 Wu, Z., Chen, X., Wang, M., Dong, J. & Zheng, Y. High-Performance Ultrathin Active Chiral Metamaterials. *ACS Nano* **12**, 5030-5041, (2018).

9 Wang, M. *et al.* Dark-Exciton-Mediated Fano Resonance from a Single Gold Nanostructure on Monolayer WS_2_ at Room Temperature. *Small* **15**, 1900982, (2019).

10 Zengin, G. *et al.* Approaching the strong coupling limit in single plasmonic nanorods interacting with J-aggregates. *Scientific Reports* **3**, 3074, (2013).

11 Sokoloff, J. P. *et al.* Transient oscillations in the vicinity of excitons and in the band of semiconductors. *Physical Review B* **38**, 7615-7621, (1988).

12 Fluegel, B. *et al.* Femtosecond Studies of Coherent Transients in Semiconductors. *Physical Review Letters* **59**, 2588-2591, (1987).

13 Lange, C. *et al.* Ultrafast control of strong light–matter coupling. *New Journal of Physics* **20**, 013032, (2018).

14 Rodek, A. *et al.* Local field effects in ultrafast light–matter interaction measured by pump-probe spectroscopy of monolayer MoSe_2_. *Nanophotonics* **10**, 2717-2728, (2021).

15 Park, J.-S. & Joo, T. Coherent interactions in femtosecond transient grating. *The Journal of Chemical Physics* **120**, 5269-5274, (2004).

16 Lietard, A., Hsieh, C.-S., Rhee, H. & Cho, M. Electron heating and thermal relaxation of gold nanorods revealed by two-dimensional electronic spectroscopy. *Nature Communications* **9**, 891, (2018).

17 Tang, Y. *et al.* Ultrafast response of a hybrid device based on strongly coupled monolayer WS_2_ and photonic crystals: the effect of photoinduced coulombic screening. **14**, 1900419, (2020).

18 Kumar, N. *et al.* Exciton-exciton annihilation in MoSe_2_ monolayers. *Physical Review B* **89**, 125427, (2014).

19 Sim, S. *et al.* Exciton dynamics in atomically thin MoS_2_ Interexcitonic interaction and broadening kinetics. *Physical Review B* **88**, 075434, (2013).

20 Antosiewicz, T. J., Apell, S. P. & Shegai, T. Plasmon–Exciton Interactions in a Core–Shell Geometry: From Enhanced Absorption to Strong Coupling. *ACS Photonics* **1**, 454-463, (2014).

21 Houdré, R. *et al.* Saturation of the strong-coupling regime in a semiconductor microcavity: Free-carrier bleaching of cavity polaritons. *Physical Review B* **52**, 7810-7813, (1995).

22 Rhee, J. K., Citrin, D. S., Norris, T. B., Arakawa, Y. & Nishioka, M. Femtosecond dynamics of semiconductor-microcavity polaritons in the nonlinear regime. *Solid State Communications* **97**, 941-946, (1996).

23 Moody, G. *et al.* Intrinsic homogeneous linewidth and broadening mechanisms of excitons in monolayer transition metal dichalcogenides. *Nature Communications* **6**, 8315, (2015).

24 Barachati, F. *et al.* Interacting polariton fluids in a monolayer of tungsten disulfide. *Nature Nanotechnology* **13**, 906-909, (2018).

25 Kravtsov, V. *et al.* Nonlinear polaritons in a monolayer semiconductor coupled to optical bound states in the continuum. *Light: Science & Applications* **9**, 56, (2020).

26 Emmanuele, R. P. A. *et al.* Highly nonlinear trion-polaritons in a monolayer semiconductor. *Nature Communications* **11**, 3589, (2020).

27 Tan, L. B. *et al.* Interacting Polaron-Polaritons. *Physical Review X* **10**, 021011, (2020).

28 Stepanov, P. *et al.* Exciton-Exciton Interaction beyond the Hydrogenic Picture in a MoSe_2_ Monolayer in the Strong Light-Matter Coupling Regime. *Physical Review Letters* **126**, 167401, (2021).

29 Zhang, L. *et al.* Van der Waals heterostructure polaritons with moiré-induced nonlinearity. *Nature* **591**, 61-65, (2021).

30 Gu, J. *et al.* Enhanced nonlinear interaction of polaritons via excitonic Rydberg states in monolayer WSe_2_. *Nature Communications* **12**, 2269, (2021).

31 Datta, B. *et al.* Highly non-linear interlayer exciton-polaritons in bilayer MoS_2_. arXiv:2110.13326 (2021).

32 Wang, S. *et al.* Limits to Strong Coupling of Excitons in Multilayer WS_2_ with Collective Plasmonic Resonances. *ACS Photonics* **6**, 286-293, (2019).

33 Erkensten, D., Brem, S. & Malic, E. Exciton-exciton interaction in transition metal dichalcogenide monolayers and van der Waals heterostructures. *Physical Review B* **103**, 045426, (2021).

34 Boulesbaa, A. *et al.* Ultrafast Dynamics of Metal Plasmons Induced by 2D Semiconductor Excitons in Hybrid Nanostructure Arrays. *ACS Photonics* **3**, 2389-2395, (2016).

35 Chen, Y.-H. *et al.* Bandgap control in two-dimensional semiconductors via coherent doping of plasmonic hot electrons. *Nature Communications* **12**, 4332, (2021).

36 Khurgin, J. B. Fundamental limits of hot carrier injection from metal in nanoplasmonics. *Nanophotonics* **9**, 453-471, (2020).
